# Evaluation of thermoregulation of different pine organs in early spring and estimation of heat reward for the western conifer seed bug (*Leptoglossus occidentalis*) on male cones

**DOI:** 10.1371/journal.pone.0272565

**Published:** 2022-08-04

**Authors:** Ryotaro Kitajima, Osamu Matsuda, Koji Mastunaga, Ryotaro Hara, Atsushi Watanabe, Atsushi Kume

**Affiliations:** 1 Department of Agro-environmental Sciences, Faculty of Agriculture, Kyushu University, Fukuoka, Japan; 2 Department of Biology, Faculty of Science, Kyushu University, Fukuoka, Japan; 3 Kyushu Regional Breeding Office, Forest Tree Breeding Center, Forestry and Forest Product Research Institute, Forest Research and Management Organization, Koshi, Kumamoto, Japan; USDA Forest Service Southern Research Station, UNITED STATES

## Abstract

The western conifer seed bug (WCSB, *Leptoglossus occidentalis*) is a pest of many pine species and is invasive worldwide. WCSB directly and indirectly deteriorates pine nut production by sucking seeds from cones. Currently, researchers think that WCSBs search for food by a combination of cues from visible light, infrared radiation, and chemicals such as monoterpenes. Some research revealed that WCSBs prefer larger cones, and it was thought that WCSBs suck seeds from and obtain more heat on larger cones. However, in early spring, we observed that most WCSBs gathered on male cones rather than on female cones and young cones. We hypothesized that male pine cones were warmer than female cones and needles, and WCSBs sucking male cones may receive more heat. To test these hypotheses, we measured spectral reflectance with a hyperspectral sensor and temperature of pine organs with tiny thermocouples, and the data were analyzed by a heat budget model. Our results revealed that male cones were significantly warmer and more reflective than female cones and needles, which may attract WCSBs. These results supported our hypothesis that WCSBs on male cones were warmer than those on other organs. This study will help further understanding of WCSBs and the adaptive value of pine cone colors.

## Introduction

The western conifer seed bug (WCSB, *Leptoglossus occidentalis*) is native to North America and a pest that affects pine nut production by sucking seeds from cones [1–4]. The damage of WCSB has been investigated in many pine species, for instance, *Pinus ponderosa* [5], *Pinus monticola* [6], *Pinus contorta* [7] and *Pinus pinea* [8]. Because those studies revealed the serious damage and WCSBs are spreading worldwide [9, 10] development of methods to control WCSBs is needed [11]. Adult WCSBs can overwinter in litter or buildings, and emerge in spring [12]. These overwintering WCSBs suck developing male cones and make them stunted [12]. It is also known that WCSBs prefer pine cones. Cone preference is not related to tree height or density of seed cones, but to the size of seed cones, with preference for larger cones [13, 14]. Additionally, a monoterpene profile difference was detected between preferred and non-preferred seed cones [13]. Furthermore, the WCSB has an organ to detect infrared radiation and is attracted by infrared radiation [15]. Although several studies proposed search cues used by WCSBs, the effectiveness of each does not seem to be definitive. Currently, it is thought that WCSBs search for seed cones by a combination of shortwave light, infrared radiation, and chemical cues [13].

The body temperature of insects is important because they are poikilotherms and strongly affected by external conditions. If the temperature is low, their activity is limited and they cannot fly. For example, bumblebees (*Bombus*) can fly in cold temperatures in early spring by warming up their thoracic temperature [16, 17]. Similar phenomena have been investigated in dragonflies, whose optimal thoracic temperature is approximately 40°C [18]. Cicadas, which belong to the same order as *L*. *occidentalis*, regulate their body temperature by metabolic generation and solar radiation [19]. Although it is not known whether WCSBs have an optimal temperature, it is possible that early spring is cold for WCSBs.

*Pinus thunbergii* belongs to Pinaceae, and all *Pinus* species are wind-pollinated. Because the cones are wind-pollinated, there is no need to attract pollinators with cone color. However, *P*. *thunbergii* exhibits different colors during early spring when its pollens are released and caught based on anthocyanin content [20], with yellow male cones and red female cones. Although the reason why female cones are red has been discussed [21–23], the actual cause is still unclear. In addition, no studies have focused on the color difference between male and female cones.

A heat budget (energy balance) model can physically estimate the temperature of an object and be used for living organisms [24]. For small objects such as WCSBs, heat storage can be ignored; therefore, WCSB input and output energy can be considered balanced. Such models can be applied to insects and small plant organs to evaluate the effects of color and morphology [24].

**Fig 1:**
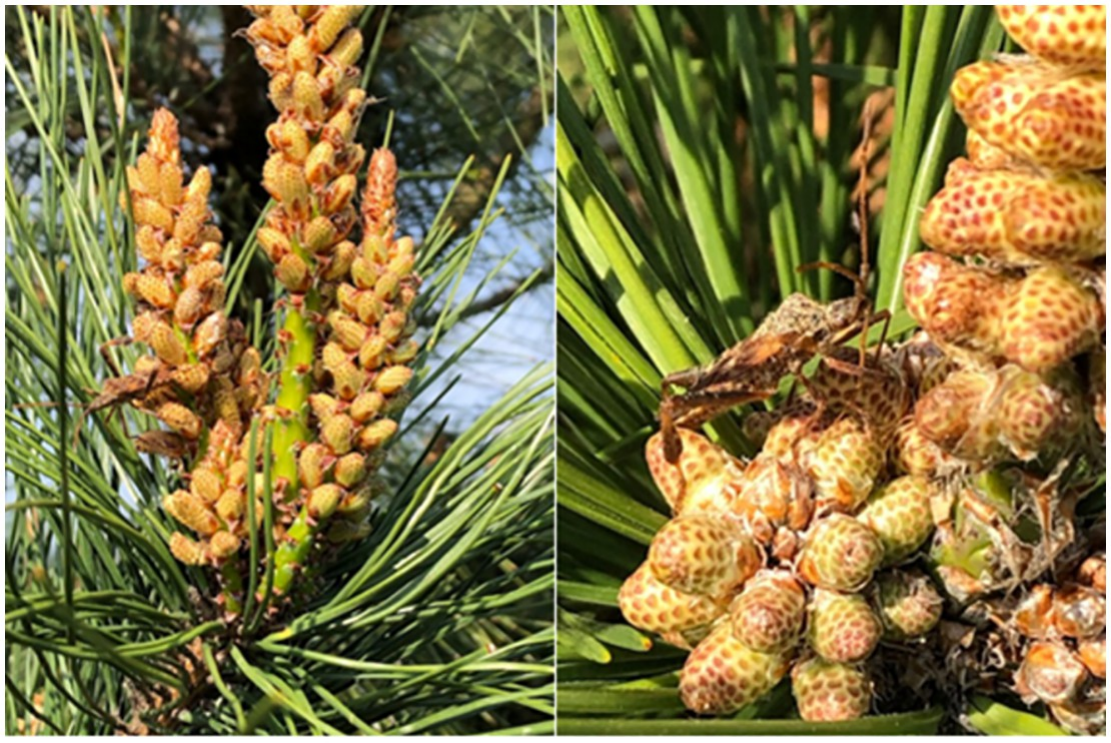
Western conifer seed bug (WCSB) holding on to male cones. These pictures were taken on 8 April 2021.

At a pine tree garden in Kumamoto Prefecture, Japan, most adult WCSBs were observed on developing male cones in early spring. Because WCSBs can detect infrared radiation, we hypothesized that male cones were warmer than needles and female cones (Fig 1). Furthermore, WCSBs may keep body temperature high on male cones. Therefore, we investigated the difference in temperature and spectral reflectance among pine organs relative to their thermoregulation, and estimated the difference in temperature between WCSBs on different pine organs using a heat budget model. This study can provide insight that may have biological application; for example, pine cone thermoregulation and an effective heat budget model may be useful for controlling WCSBs.

## Materials and methods

### Study site and plant materials

We conducted our study at the experimental crossing garden of Forest Tree Breeding Center Kyushu Regional Breeding Office (Kumamoto, Japan, 32.8816°N, 130.7357°E) on 1 April 2021, and the roof of West 5 Building of Kyushu University (Fukuoka, Japan, 33.5942°N, 130.2146°E) on 19 January 2021. In Kumamoto, *P*. *thunbergii* grafted in 2015 was in bloom on 1 April. The trees were pruned to a height of approximately 3 m in late fall. Male cones, female cones, and needle leaves were sampled, and the spectral reflectance was measured the next day.

### Spectral reflectance measurement

Spectral reflectance was measured by a pushbroom scanning system. The imaging system was similar to those in previous studies [25, 26] except that the light source consisted of a pair of DC halogen lamps. A line-scanning hyperspectral camera (VNIR-200R, Themis Vision Systems, Bay St Louis, USA) sensitive to visible to near-infrared wavelengths (450–980 nm) was used. The camera was equipped with a silicon charge-coupled-device detector and was capable of acquiring 1,392 × 1,040 pixel images with 12-bit digitization and at 1.3-nm spectral resolution. The reflectance was obtained based on a white filter paper placed beside the stage as a reference surface. The visible (450–780 nm) and near-infrared (780–980 nm) reflectance of the filter paper were 93.0% and 97.8%, respectively, and the obtained reflectance was calibrated with a PTFE standard reflector (SphereOptics GmbH, Uhldingen, Germany). Four dead bodies of WCSBs, which were stored in -40°C for two months, were captured. After capturing the images, the area of the WCSB was manually extracted from each image. The spectral reflectance of each WCSB was determined by averaging the spectral reflectance of each pixel.

### Measurements

To establish a heat budget model, environmental conditions were measured on a sunny day, 1 April 2021. Air temperature and humidity were measured by Thermo Recorder (TR-72wb, T&D Co., Tokyo, Japan) at 1.9 m above ground. Shortwave radiation was measured by a pyranometer (SP-110-SS, Apogee Instruments, Inc., Logan, USA). Temperatures of male cones, female cones, and needles in the same shoots were measured with thermocouples (ϕ = 0.12 mm). Sample sizes were three for male and female cones and two for needles. Wind speed and ground temperature were estimated from micrometeorological measurements. The obtained organ temperatures were used for further accuracy testing.

**Fig 2:**
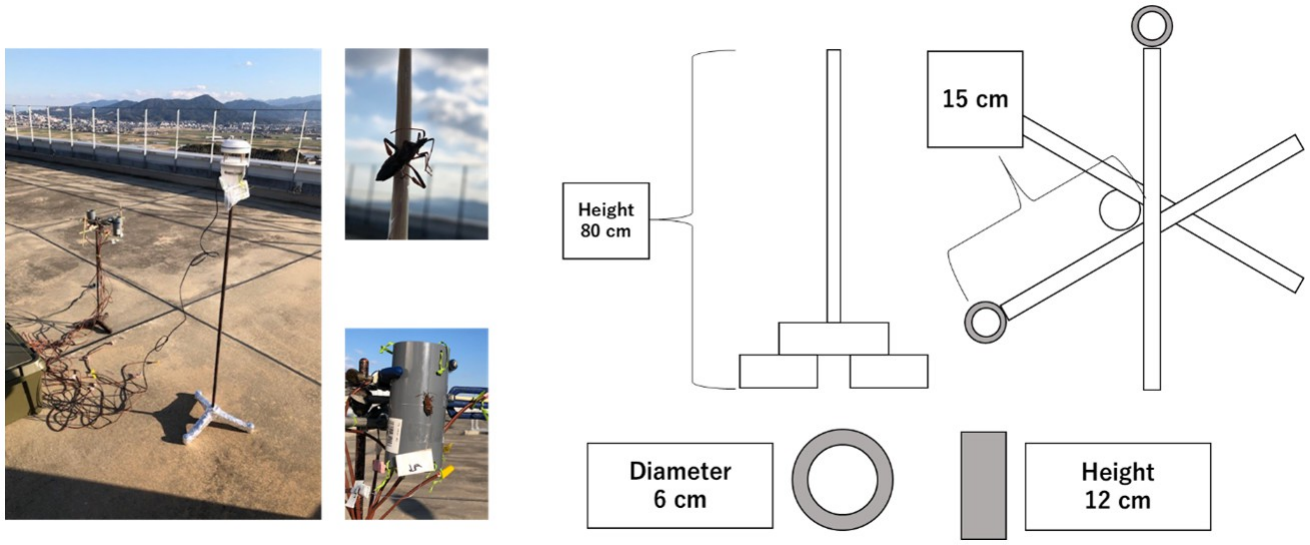
Instruments for testing accuracy of the heat budget model. Thermocouples were attached to dead WCSBs and tips of 15-cm arms. A PVC tube was used to simulate the effect of obstacles near WCSBs.

WCSB body temperature was measured on 19 January 2022 on the roof of Kyushu University West 5 Building using two dead WCSBs. The thermocouples were attached to each dead WCSB body with cyanoacrylate adhesive, and to a polyvinyl chloride (PVC) tube with tape. The detailed size and structure are shown in Fig 2.

Each of the two WCSBs was oriented horizontally and vertically. The PVC tube was used to simulate the effect of objects near the WCSB and its shortwave reflectance was set to 0.2. Air temperature and humidity were measured by Thermo Recorder. Wind speed was measured using a sonic anemometer (ATMOS-22, METER Group, Inc., Pullman, USA) at 1.2 m above ground. All data were recorded once per 30 s and averaged over 10 min. Heat budget model accuracy was tested by root mean square error (RMSE), bias, and determination coefficients.

The heat budget model also required shortwave absorptance. This was obtained by integration of spectral reflectance and spectral radiation. Because spectral characteristics differ between sunny and cloudy days [27], absorption differs depending on the weather. Spectral radiation was measured on the roof of West 5 Building of Kyushu University (Fukuoka, Japan) with an MS-711 spectroradiometer (EKO Instruments Co., Ltd., Tokyo, Japan) with a rotating shadow band (RSB-01, EKO Instruments Co., Ltd., Tokyo, Japan). Because it was sunny on 1 April 2021, spectral radiation on a sunny day was used to calculate absorptance.

### Heat budget formulae

**Fig 3:**
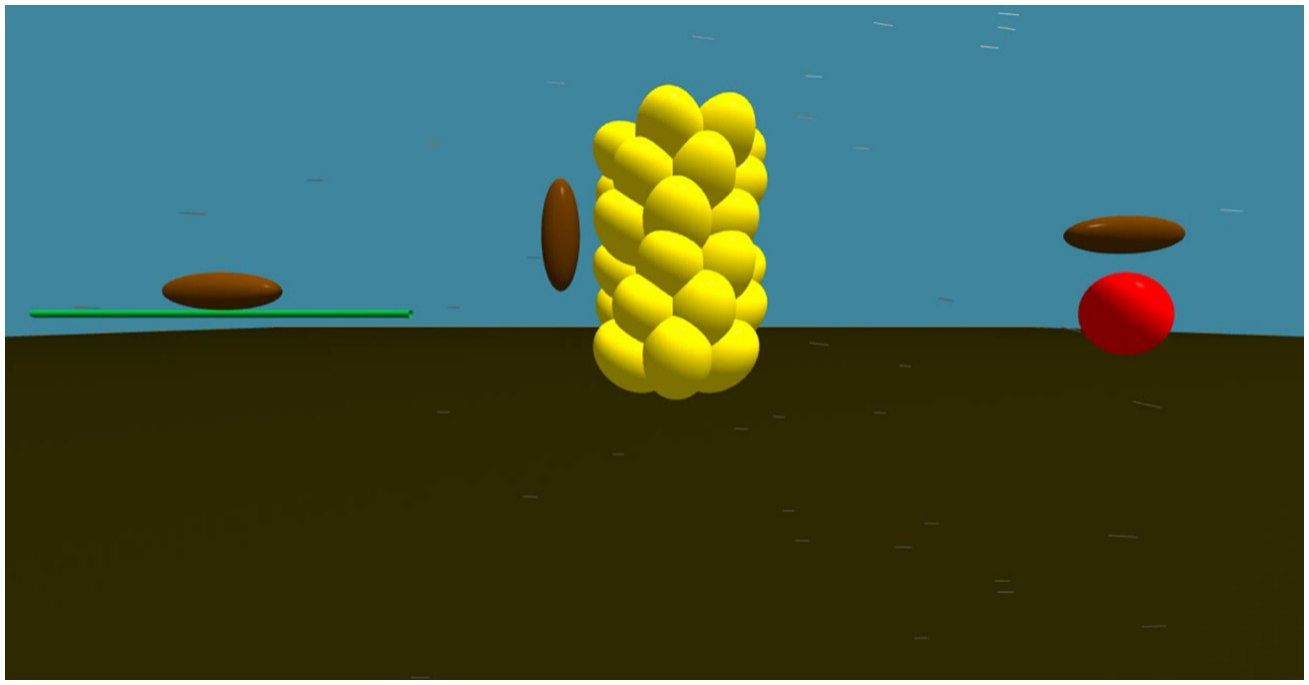
Assumptions for heat budget estimation of WCSBs. Left: WCSB on a needle. Center: WCSB on male cones. Right: WCSB on a female cone.

We made a heat budget model from the following formulae [24]. We simulated the surface temperature of WCSBs on a needle, a male cone inflorescence, and a female cone (Fig 3). To calculate heat budget, the WCSB was approximated as an ellipsoid whose long and short axes were 15 mm and 5 mm, respectively, and its leg length was set to 5 mm. The female cone was also approximated as an ellipsoid, whose long and short axes were 11 mm and 8 mm, respectively. The needle was approximated as a cylinder whose radius and length were 1 mm and 100 mm, respectively. Male cone inflorescence was approximated as a cylinder whose radius and height were set to 11 mm and 45 mm, respectively.

Radiation input was obtained from the following formulae.

$$R_{in}=R_{sd}\left(1-\alpha_{p}\right)\left(p_{dir}\;F_{d}+p_{diff}\left(F_{sky}+a_{object}\;F_{object}+a_{ground}\;F_{ground}\right)\right)+\left({F_{sky}\;R}_{ld}+F_{ground}\;R_{lu}+F_{object}\;R_{lobject}\right)$$ (1)

$$R_{ld}=\sigma T_{g}^{4}$$ (2)

$$R_{lu}=\sigma {\left(T_{a}-20\right)}^{4}$$ (3)

$$R_{lobject}=\sigma T_{object}^{4}$$ (4)

where *R*_*in*_ means the total radiation input (W m^−2^), *R*_sd_ is shortwave radiation, *α*_p_ is reflectance obtained from the integration of spectral reflectance and spectral radiation (W m^−2^), and *p*_dir_ and *p*_diff_ represent the ratio of direct radiation and diffuse radiation in total radiation [28], respectively. *α*_ground_ and *α*_object_ are shortwave reflectance of the ground and object, respectively. *R*_ld_, *R*_lu_, and *R*_lobject_ are longwave radiation from the sky, ground, and object (W m^−2^), respectively. *F*_d_, *F*_ground_, and *F*_object_ are view factors of the sky, ground, and object, respectively. σ is the Stefan–Boltzmann constant. *T*_g_ and *T*_a_ are ground and air temperature (K), longwave radiation of the sky was estimated from assuming sky as a blackbody cooler than atmosphere by 20 K [29].


$$R_{net}=R_{in}-\sigma T_{s}^{4}$$ (5)

*R*_net_ is net radiation obtained from subtracting longwave radiation from input radiation. *T*_s_ is the temperature of the target object.

$$R_{net}=H+lE$$ (6)

$$H=c_{p}\times 1.4\times 0.135\times \sqrt{\frac{u}{d}}\left(T_{s}-T_{a}\right)$$ (7)

$$lE=l\times 1.4\times 0.147\times \sqrt{\frac{u}{d}}\frac{e\left(T_{s}\right)-q_{a}}{P_{a}}$$ (8)

where *H* is a sensible heat exchange (*H*: W m^−2^), *c*_p_ is specific heat at constant pressure of atmosphere (J mol^-1^ K^-1^), *u* is wind speed (m s^−1^), *d* is the characteristic length (m), *lE* means latent heat exchange (*lE*: W m^−2^), *l* is latent heat of vaporization (kJ mol^−1^), and *E* is water vapor flux density (mol m^−2^). e(*T*_*s*_) is saturated vapor pressure at *T*_s_, *q*_a_ is water vapor pressure, and P_a_ is atmospheric pressure. In this study, the heat storage and latent heat exchange from the WCSB surface were ignored; therefore, *R*_net_ was dispersed only by sensible heat exchange (*H*). For *P*. *thunbergii* organ temperature, *R*_net_ was dispersed by both latent heat and sensible heat.

#### Determination of view factor

*F*_*d*_ represents the view factor of objects for direct radiation and was calculated from the following formulae (9) for vertical, (10) for horizontal and (11) for shape ratio,

$$F_{d}=\frac{\sqrt{1+\left(x^{2}-1\right)\;\text{sin}\;\theta }}{2x+\frac{\left(2{\;\text{sin}}^{-1}\sqrt{1-x^{2}}\right)}{\sqrt{1-x^{2}}}}$$ (9)

$$F_{d}=\frac{\sqrt{1+\left(x^{2}-1\right)\text{cos}\;\theta }}{2x+\frac{\left(2\;{\text{sin}}^{-1}\sqrt{1-x^{2}}\right)}{\sqrt{1-x^{2}}}}$$ (10)

$$x=\frac{a}{b}$$ (11)

where *θ* represents solar elevation, and *a* and *b* represent the long and short axes of ellipsoids, respectively.

Other view factors (*F*_sky_, *F*_ground_, and *F*_object_) were different depending on kind of objects and distances from the objects. They were estimated using TouchDesigner 2020.25380 (Derivative Inc., Toronto, Canada), a 3D modeling software program, and ImageJ 1.53K [30].

We prepared “Tube SOP” as the needle and male inflorescence and “Sphere SOP” as the female cone in TouchDesigner. “Camera COMP” was placed 5 mm away from the objects. To obtain the scene from Camera COMP, “Render TOP” was used and set to “Fish-Eye (180)” to obtain 180 FOV pictures. Obtained pictures were output by “Movie File Out TOP” and input in ImageJ. The area ratio of each object (sky, ground, and objects) was obtained by ImageJ’s threshold feature.

In view factors for *P*. *thunbergii*, *F*_object_ was set to 0, and *F*_sky_ and *F*_ground_ were set to 0.5. *F*_d_ was obtained from formula (9).

### Statistical analysis

A time-based analysis was conducted along with analysis of variance (ANOVA) in R 4.0.2 [31]. Heat budget calculation was conducted in R 4.0.2 [31] with the packages “suncalc” [32] and “nleqslv” [33].

## Results

### Spectral reflectance

**Fig 4:**
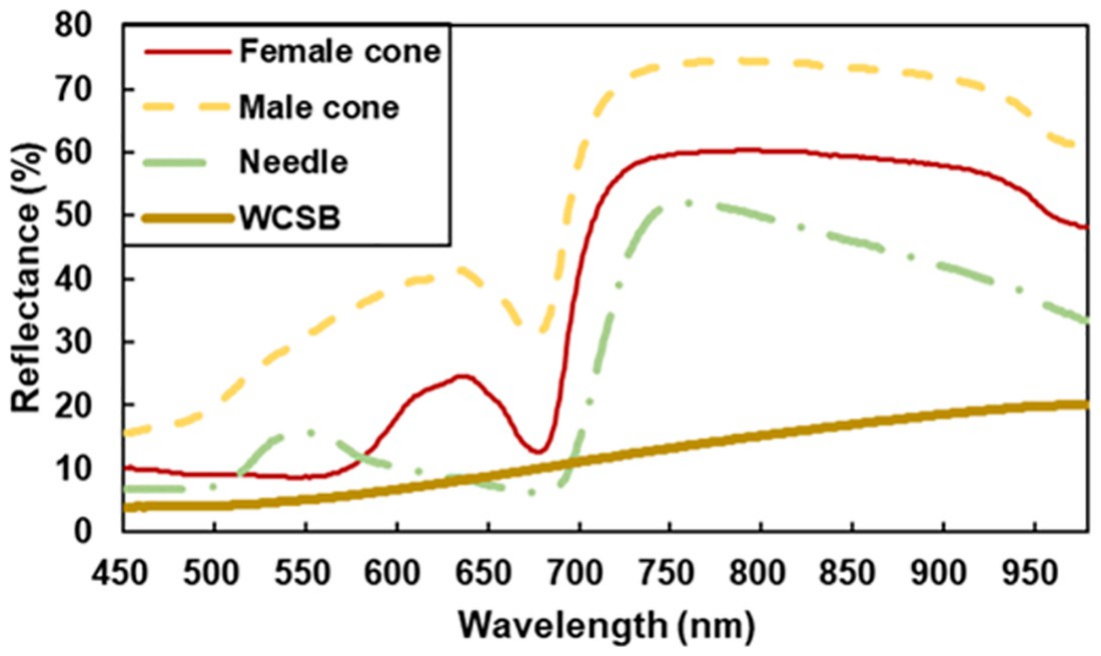
Spectral reflectance of pine plant organs and dead bodies of WCSB. Lines represent mean values.

Fig 4 shows the spectral reflectance of *P*. *thunbergii* organs and *L*. *occidentalis*. Among the three *P*. *thunbergii* organs, the male cone had the highest reflectance. Female cones and needles had low reflectance but relatively high reflectance in the red range (~620–750 nm) for female cones and in the green range (~500–565 nm) for needles. *Leptoglossus occidentalis* had low reflectance over the measured wavelength range and relatively high reflectance at long wavelengths.

### Field measurements

**Fig 5:**
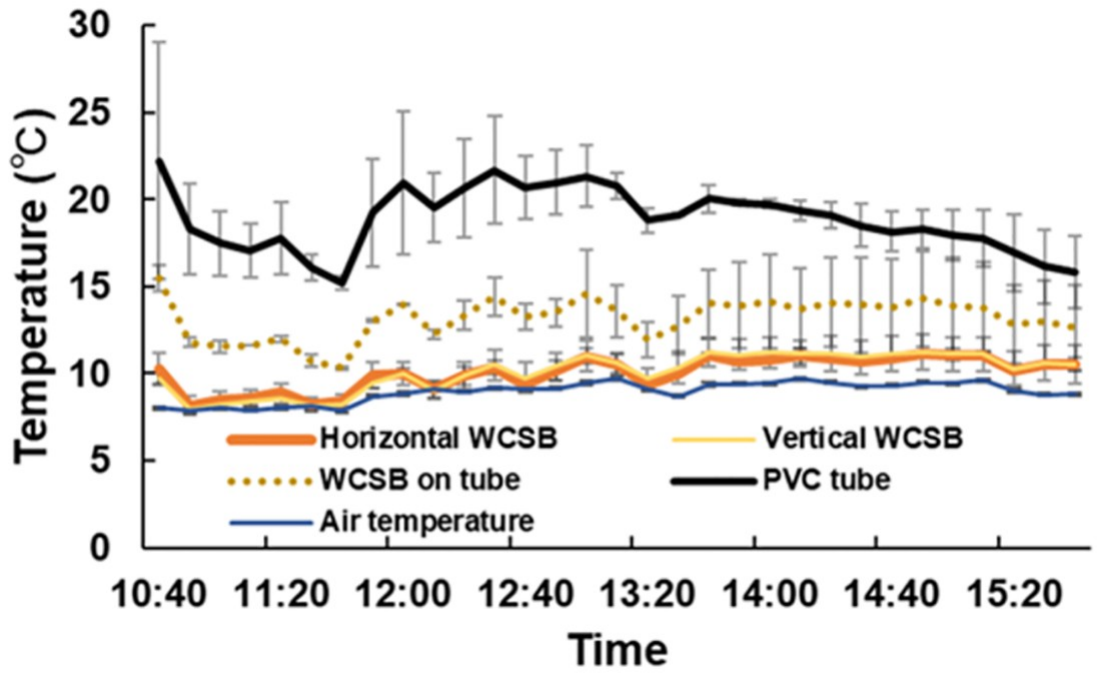
Temperature measurements on the roof of West 5 Building of Kyushu University on 19 January 2022. Lines represent mean values and error bars represent standard deviation.

**Fig 6:**
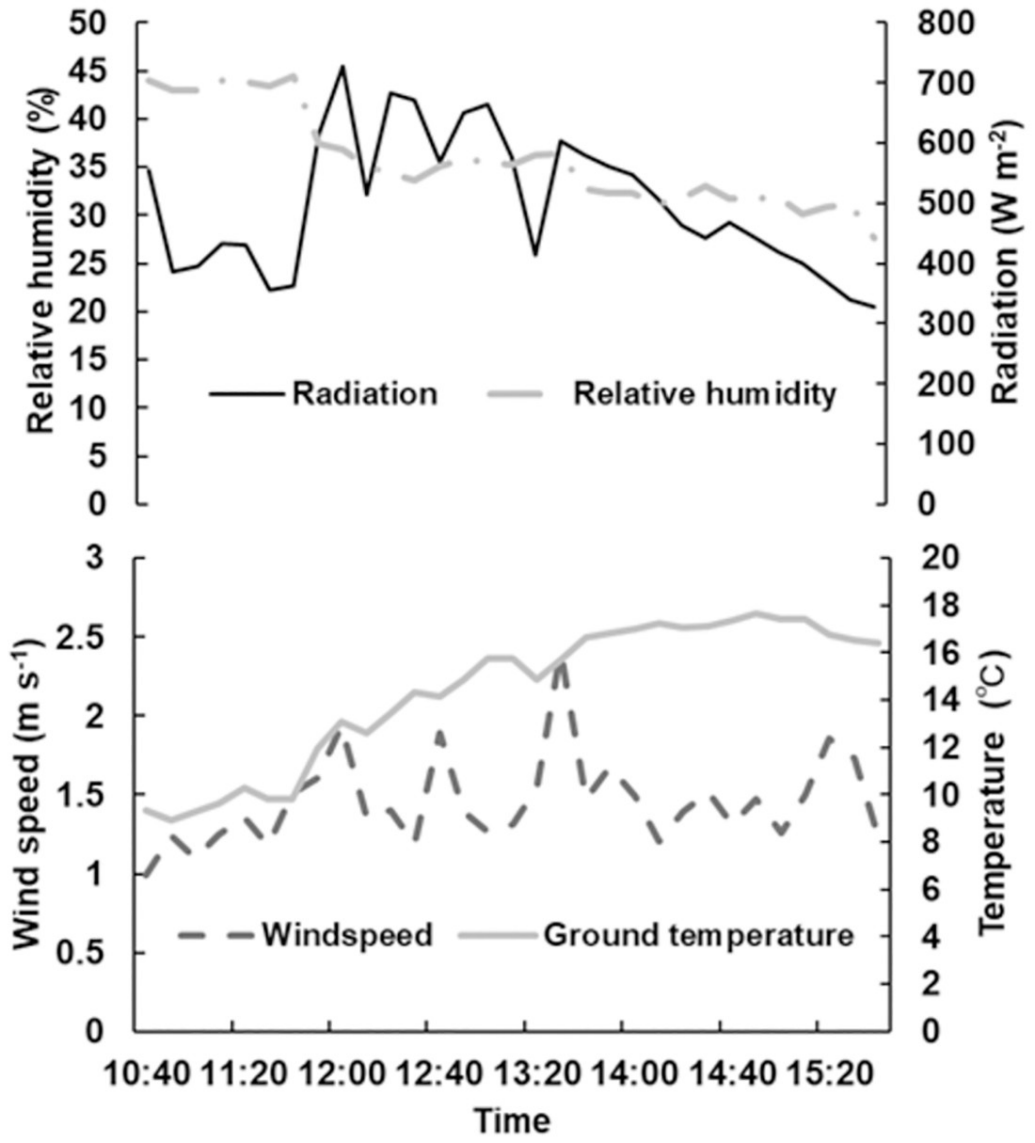
Environmental conditions at the roof of West 5 Building of Kyushu University on 19 January 2022. The top graph shows radiation and relative humidity, and the bottom graph shows wind speed and ground temperature.

Figs 5 and 6 show the measurements recorded at Kyushu University on 19 January 2022. The results showed that WCSBs on the PVC tube were warmer than other WCSBs. WCSBs on the PVC tube were warmer than the air temperature by approximately 5°C. These values were further used for the heat budget model and accuracy test.

**Fig 7:**
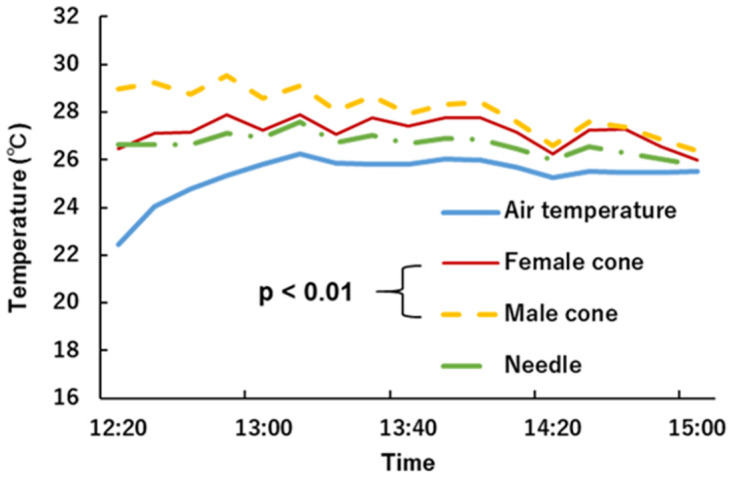
Temperature measurements of *Pinus thunbergii*. Asterisks indicate a significant difference between male and female cones.

**Fig 8:**
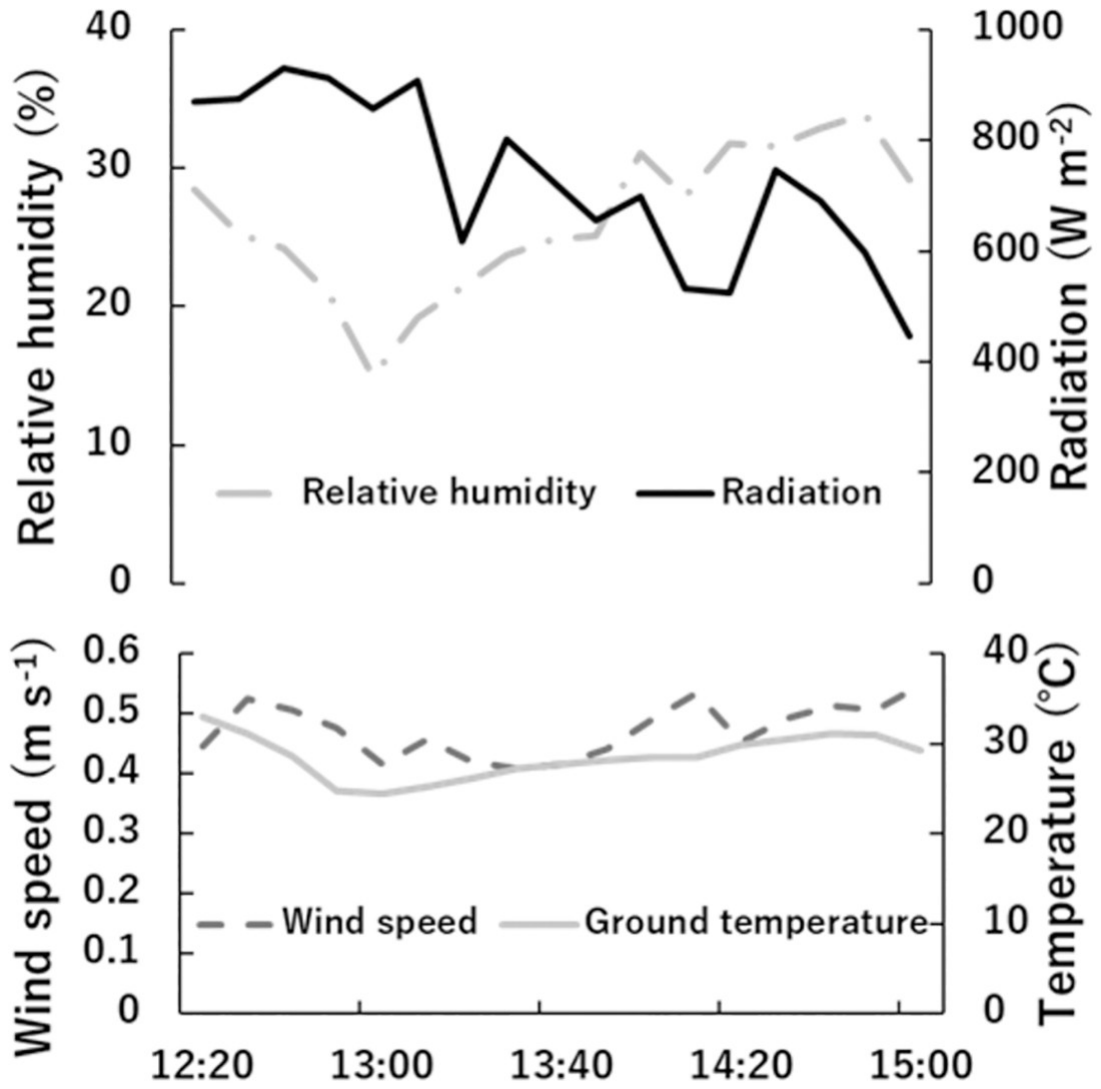
Environmental conditions in the field on April 1^st^, 2021. The top graph shows relative humidity and radiation measurements. The bottom graph shows the estimation result of wind speed at 1.9 m above ground and ground temperature. These results were used to calculate heat budget.

Figs 7 and 8 show the environmental conditions and organ temperature measurements in the field on 1 April 2021 at the tree garden. Male cone temperature was the highest of the three organs, and the temperatures of the other two organs were similar to each other. The ANOVA results showed that there was a significant difference between male and female cone temperatures. The environmental conditions were used for heat budget calculation.

### View factors

**Fig 9:**
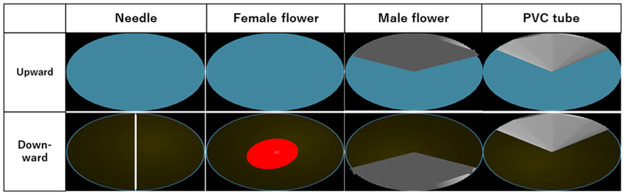
Views from WCSBs on needles, female cones, male cones, and PVC tubes.

Fig 9 shows the views from WCSBs on different objects, and Table 1 shows the obtained view factors from Fig 9. Excluding male cones and the PVC tube, the upward view factors were dominated by sky. On male cones, the area occupied by the male cone was approximately one-third of the area. Thus, view factors were influenced by object size. These values were used for heat budget calculations.

**Table 1:** Detailed values of view factors (*F*) for heat budget calculation.

		Sky	Ground	Object
Female	Upward	0.500	0.000	0.000
	Downward	0.013	0.415	0.072
Male	Upward	0.347	0.000	0.153
	Downward	0.009	0.344	0.147
Needle	Upward	0.500	0.000	0.000
	Downward	0.013	0.466	0.021
Tube	Upward	0.341	0.000	0.159
	Downward	0.009	0.328	0.162

For example, *F*_sky_ for WCSBs on male cones can be seen under the “Male” row and “Sky” column.

### Estimation of male and female cone temperatures

The accuracy of temperature estimation for the male and female cones was 2.04°C for RMSE and 0.41°C for bias. To confirm the effect of different colors, we estimated the temperature using objects with female cone color and male cone size, and male cone color with female cone size under the microclimate conditions recorded on 1 April 2021. The temperature of the object with female cone color and male cone size was 29.0°C on average (31.0°C maximum). These values were higher than those of the estimated male cone temperature by 0.7°C on average (0.9°C maximum). The temperature of the object with male cone color and female cone size was 27.4°C on average (28.8°C maximum). These values were lower than those of the estimated female cone temperature by 0.4°C on average (0.5°C maximum).

### Estimation of *L*. *occidentalis* temperature

**Fig 10:**
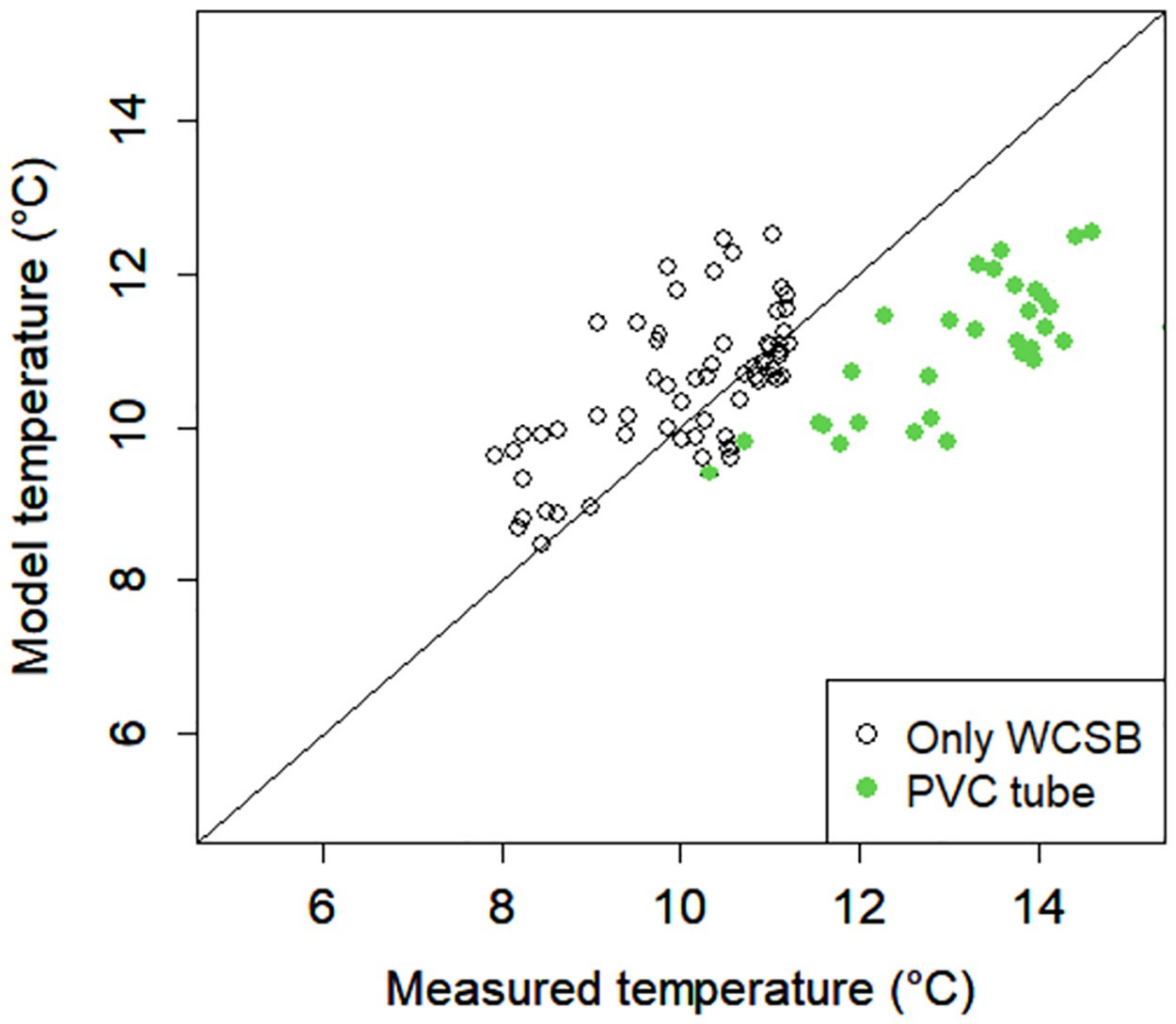
Accuracy test of the heat budget model that estimated WCSB temperature. Root mean square error, bias, and determination coefficient were 1.52°C, –0.39°C, and 0.341, respectively.

Fig 10 shows the accuracy of the model estimating WCSB temperature. RMSE, bias, and determination coefficient were 1.52°C, –0.39°C, and 0.341, respectively. However, the WCSB temperature near the PVC tube was underestimated, and RMSE, bias, and determination coefficient were 2.26°C, –2.12°C, and 0.559, respectively.

**Fig 11:**
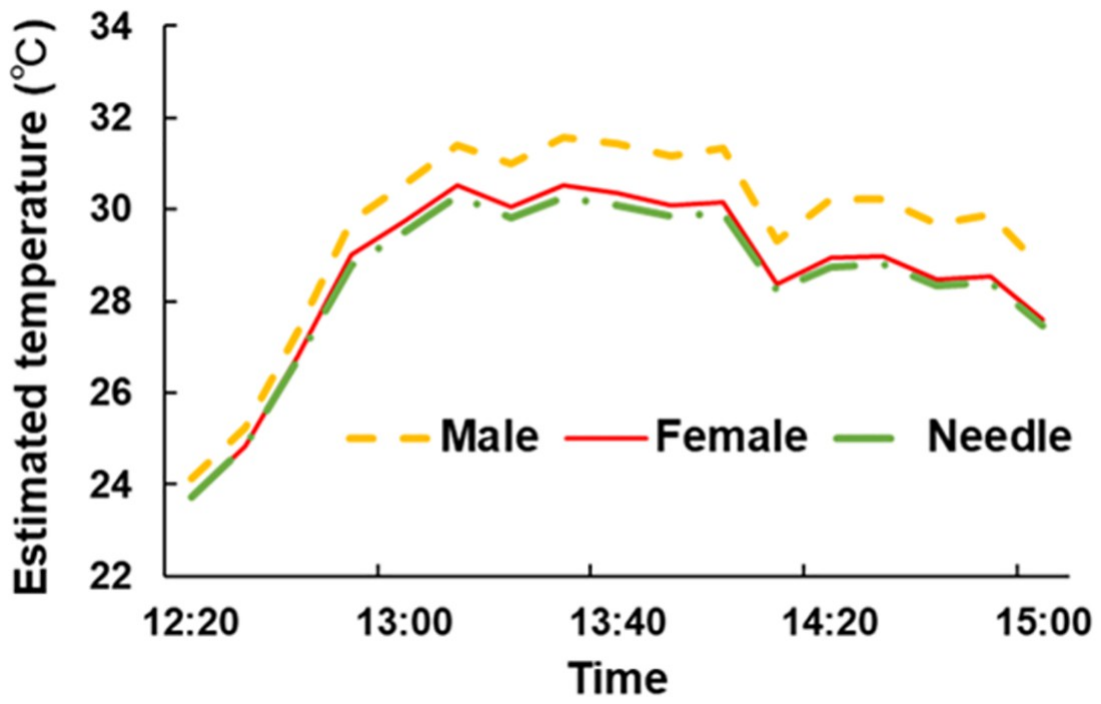
Estimated temperature of WCSBs on different plant organs. This estimation was conducted using environmental data measured on 1 April 2021.

Under the environmental conditions on 1 April 2021, WCSBs on male cones were warmer than those on the other two organs (Fig 11). The mean temperature difference was approximately 1°C during this period. The temperatures of WCSBs on needles and female cones were approximately the same.

## Discussion

Clarifying WCSB host tree preference is important for controlling WCSB populations. Because of their importance, previous researchers tried to identify and hypothesize visible light cues [14], infrared radiation cues [15], and volatile chemical compound cues [13]. However, some researchers could not find differences in certain cues, such as infrared radiation from cones [13]. WCSB host preference has puzzled many researchers and is still unclear [8, 13–15]. The present study provides insight based on the thermoregulation strategy of pine and micrometeorology.

There is a potential trade-off between thermoregulation and maximizing offspring production in pine male cones, and male cone color may be adaptive. Because Pinaceae species are anemophilous, the color of cones would not need to attract pollinators. In female cones, this has been discussed in the context of three hypotheses: thermoregulation, photoprotection, and anti-herbivory [21–23, 34]. However, there are no studies that focused on male cone color and its adaptive implications. Pine trees need to produce a lot of pollen and male cones [35]. Large male inflorescences are attached, and large and dense inflorescences are inefficient in exchanging heat with air (Eqs (7) and (8)). Although pine pollen is resistant to temperature and dryness once released, male cones before releasing pollens are considered sensitive to high temperatures, and over 30°C is potentially harmful [36, 37]. Therefore, the higher reflectance in male cones may indicate a heat-blocking strategy to reduce the amount of radiation input. The pigmentation with anthocyanins may be advantageous even in male cones because the female red color is thought to provide some protection against shortwave radiation, especially UV-B radiation. However, an object with female cone color and male cone size was estimated to exceed 30°C; therefore, the simultaneous pigmentation protection may cause heat stress and be lethal for male cones. Therefore, such protection by anthocyanins could be a “privilege” only for female cones because of its high cooling efficiency, and male cones could be forced to have high reflectance to not get too warm.

In early spring, WCSB are more commonly found on male cones. There can be several hypotheses; heat rewarding, which is discussed in this study, nutrient difference between male and female cones [38, 39], the conspicuity of WCSB on female cone [40] and so on. Our result cannot show definitively which hypothesis is true but can support the heat rewarding hypothesis. Male cones have higher reflectance for shortwave radiation (Fig 4) and higher temperatures (Fig 7). Objects with high temperature emit more infrared radiation, and WCSBs may be able to detect it. In addition, higher temperatures result in greater release of volatile compounds such as monoterpenes. In terms of nutrients, pine pollen is rich in sugar and N contents [38, 39], which is similarly true of male cones before releasing pollen. Therefore, male cones may be more conspicuous and attractive to WCSBs because of their high reflectance, high temperature, and high concentration of volatile compounds and nutrients.

WCSBs suck and get heat from male cones. WCSBs on male cones were warmer than individuals on needles or female cones, and the mean temperature difference was estimated to be at least 1°C. Here, we emphasize that this model does not consider the differences in the characteristic length of organs. Therefore, the difference in temperature was only caused by difference in radiation balance. However, Fig 10 shows that there was a gap between measured and estimated air temperatures that cannot be explained by the radiation balance. Large objects obstruct the wind, which results in less heat exchange. The gap in Fig 10 can be explained by this hindered heat exchange. Fig 10 shows that WCSBs on male cones can be 3°C warmer than individuals on needles and female cones.

## Conclusion

This study quantified the body temperature of WCSBs on different pine organs and addressed pine cone thermoregulation. A heat budget model based on field measurements was applied to estimate temperatures of WCSBs and pine organs. Field measurements showed that male cones were significantly warmer and more reflective than other organs, which indicates that some features of male cones may attract WCSBs. Additionally, cone colors may represent a thermoregulation strategy. The heat budget model showed that the temperatures of WCSBs on male cones on a clear day were more than 1°C higher than those of the individuals on female cones and needles. Because this model only considered the effect of the radiation balance and did not include the effects of boundary layer resistance, the actual effect of the temperature increase on male cones would be approximately 3°C. This result emphasizes the advantage of staying on male cones in early spring because WCSBs can receive heat. This study provides insight into WCSB host selection and methods for estimating the temperature environment of insects.

## Supporting information

S1 File Numerical data used in the figures. (XLSX)

S1 Text R script used for analyses (ANOVA_Pthunbergii). (R)

S2 Text R script used for analyses (Heat Budget_Pthunbergii). (R)

S3 Text R script used for analyses (Heat Budget_Loccidentalis). (R)
