# Correction: Evaluation of thermoregulation of different pine organs in early spring and estimation of heat reward for the western conifer seed bug (Leptoglossus occidentalis) on male cones

**DOI:** 10.1371/journal.pone.0305374

**Published:** 2024-06-06

**Authors:** Ryotaro Kitajima, Osamu Matsuda, Koji Matsunaga, Ryotaro Hara, Atsushi Watanabe, Atsushi Kume

The third author’s name is spelled incorrectly. The correct name is: Koji Matsunaga. The correct citation is: Kitajima R, Matsuda O, Matsunaga K, Hara R, Watanabe A, Kume A (2022) Evaluation of thermoregulation of different pine organs in early spring and estimation of heat reward for the western conifer seed bug (Leptoglossus occidentalis) on male cones. PLoS ONE 17(8): e0272565. https://doi.org/10.1371/journal.pone.0272565.
