# Supplementary material for: Evaluation of thermoregulation of different pine organs in early spring and estimation of heat reward for the western conifer seed bug (Leptoglossus occidentalis) on male cones
Source: PLoS One. 2022 Aug 4;17(8):e0272565. doi: 10.1371/journal.pone.0272565 (PMC9352051; doi:10.1371/journal.pone.0272565)
Supplement: Attachment — Submitted filename: PONE-D-22-12856_fhk.pdf [file pone.0272565.s005.pdf]

PLOS ONE

Evaluation of thermoregulation of different pine organs in early spring and estimation of heat reward for the western conifer seed bug (Leptoglossus occidentalis) on male flowers

--Manuscript Draft--

**Manuscript Number:**	PONE-D-22-12856
**Article Type:**	Research Article
**Full Title:**	Evaluation of thermoregulation of different pine organs in early spring and estimation of heat reward for the western conifer seed bug (Leptoglossus occidentalis) on male flowers
**Short Title:**	Heat reward estimation of western conifer seed bugs on male pine flowers
**Corresponding Author:**	Atsushi Kume<br>Kyushu University - Ito Campus: Kyushu Daigaku<br>Fukuoka, JAPAN
**Keywords:**	anemophilous flower; flower thermoregulation; heat budget; Leptoglossus occidentalis; Pinus thunbergii; spectral reflectance
**Abstract:**	The western conifer seed bug (WCSB, Leptoglossus occidentalis) is a pest of many pine species and is invasive worldwide. WCSB directly and indirectly deteriorates pine nut production by sucking seeds from cones. Currently, researchers think that WCSBs search for food by a combination of cues from visible light, infrared radiation, and chemicals such as monoterpenes. Some research revealed that WCSBs prefer larger cones, and it was thought that WCSBs suck seeds from and obtain more heat on larger cones. However, in early spring, we observed that most WCSBs gathered on male flowers rather than on female flowers and young cones. We hypothesized that male pine flowers were warmer than female flowers and needles, and WCSBs sucking male flowers may receive more heat. To test these hypotheses, we measured spectral reflectance with a hyperspectral sensor and temperature of pine organs with tiny thermocouples, and the data were analyzed by a heat budget model. Our results revealed that male flowers were significantly warmer and more reflective than female flowers and needles, which may attract WCSBs. These results supported our hypothesis that WCSBs on male flowers were warmer than those on other organs. This study will help further understanding of WCSBs and the adaptive value of pine flower colors.
**Order of Authors:**	Ryotaro Kitajima
	Osamu Matsuda
	Koji Mastunaga
	Ryotaro Hara
	Atsushi Watanabe
	Atsushi Kume
**Opposed Reviewers:**	
**Additional Information:**	
**Question**	**Response**
**Financial Disclosure**<br><br>Enter a financial disclosure statement that describes the sources of funding for the work included in this submission. Review the submission guidelines for detailed requirements. View published research	The authors received no specific funding for this work.

Powered by Editorial Manager® and ProduXion Manager® from Aries Systems Corporation

articles from *PLOS ONE* for specific examples.<br><br>This statement is required for submission and **will appear in the published article** if the submission is accepted. Please make sure it is accurate.<br><br>**Unfunded studies**<br>Enter: *The author(s) received no specific funding for this work.*<br><br>**Funded studies**<br>Enter a statement with the following details:<br>• Initials of the authors who received each award<br>• Grant numbers awarded to each author<br>• The full name of each funder<br>• URL of each funder website<br>• Did the sponsors or funders play any role in the study design, data collection and analysis, decision to publish, or preparation of the manuscript?<br>• **NO** - Include this sentence at the end of your statement: *The funders had no role in study design, data collection and analysis, decision to publish, or preparation of the manuscript.*<br>• **YES** - Specify the role(s) played.<br><br>* typeset	
**Competing Interests**<br><br>Use the instructions below to enter a competing interest statement for this submission. On behalf of all authors, disclose any competing interests that could be perceived to bias this work—acknowledging all financial support and any other relevant financial or non-financial competing interests.<br><br>This statement is required for submission and **will appear in the published article** if the submission is accepted. Please make sure it is accurate and that any funding sources listed in your Funding Information later in the submission form are also declared in your Financial Disclosure statement.	The authors have declared that no competing interests exist.

View published research articles from *PLOS ONE* for specific examples.<br><br>**NO authors have competing interests**<br><br>Enter: *The authors have declared that no competing interests exist.*<br><br>**Authors with competing interests**<br><br>Enter competing interest details beginning with this statement:<br><br>*I have read the journal's policy and the authors of this manuscript have the following competing interests: [insert competing interests here]*<br><br>* typeset	
**Ethics Statement**<br><br>Enter an ethics statement for this submission. This statement is required if the study involved:<br><br>• Human participants<br>• Human specimens or tissue<br>• Vertebrate animals or cephalopods<br>• Vertebrate embryos or tissues<br>• Field research<br><br>Write "N/A" if the submission does not require an ethics statement.<br><br>General guidance is provided below. Consult the submission guidelines for detailed instructions. **Make sure that all information entered here is included in the Methods section of the manuscript.**	N/A

Powered by Editorial Manager® and ProduXion Manager® from Aries Systems Corporation

**Format for specific study types**<br><br>**Human Subject Research (involving human participants and/or tissue)**<br>• Give the name of the institutional review board or ethics committee that approved the study<br>• Include the approval number and/or a statement indicating approval of this research<br>• Indicate the form of consent obtained (written/oral) or the reason that consent was not obtained (e.g. the data were analyzed anonymously)<br><br>**Animal Research (involving vertebrate animals, embryos or tissues)**<br>• Provide the name of the Institutional Animal Care and Use Committee (IACUC) or other relevant ethics board that reviewed the study protocol, and indicate whether they approved this research or granted a formal waiver of ethical approval<br>• Include an approval number if one was obtained<br>• If the study involved *non-human primates*, add *additional details* about animal welfare and steps taken to ameliorate suffering<br>• If anesthesia, euthanasia, or any kind of animal sacrifice is part of the study, include briefly which substances and/or methods were applied<br><br>**Field Research**<br><br>Include the following details if this study involves the collection of plant, animal, or other materials from a natural setting:<br>• Field permit number<br>• Name of the institution or relevant body that granted permission	
**Data Availability**<br><br>Authors are required to make all data underlying the findings described fully available, without restriction, and from the time of publication. PLOS allows rare exceptions to address legal and ethical concerns. See the PLOS Data Policy and FAQ for detailed information.	Yes - all data are fully available without restriction

Powered by Editorial Manager® and ProduXion Manager® from Aries Systems Corporation

A Data Availability Statement describing where the data can be found is required at submission. Your answers to this question constitute the Data Availability Statement and **will be published in the article**, if accepted.<br><br>**Important:** Stating 'data available on request from the author' is not sufficient. If your data are only available upon request, select 'No' for the first question and explain your exceptional situation in the text box.<br><br>Do the authors confirm that all data underlying the findings described in their manuscript are fully available without restriction?	
**Describe where the data may be found in full sentences. If you are copying our sample text, replace any instances of XXX with the appropriate details.**<br><br>• If the data are **held or will be held in a public repository**, include URLs, accession numbers or DOIs. If this information will only be available after acceptance, indicate this by ticking the box below. For example: *All XXX files are available from the XXX database (accession number(s) XXX, XXX.*).<br>• If the data are all contained **within the manuscript and/or Supporting Information files**, enter the following: *All relevant data are within the manuscript and its Supporting Information files.*<br>• If neither of these applies but you are able to provide **details of access elsewhere**, with or without limitations, please do so. For example:<br><br>*Data cannot be shared publicly because of [XXX]. Data are available from the XXX Institutional Data Access / Ethics Committee (contact via XXX) for researchers who meet the criteria for access to confidential data.*<br><br>*The data underlying the results presented in the study are available from (include the name of the third party*	All relevant data are within the manuscript and its Supporting Information files.

and contact information or URL).<br>• This text is appropriate if the data are owned by a third party and authors do not have permission to share the data.<br><br>* typeset	
Additional data availability information:	

Powered by Editorial Manager® and ProduXion Manager® from Aries Systems Corporation

Dr Emily Chenette,
Editor in Chief
PLoS ONE

30 April 2022

Dear Dr. Chenette,

We would like to submit the new manuscript entitled "Evaluation of thermoregulation of different pine organs in early spring and estimation of heat reward for the western conifer seed bug (*Leptoglossus occidentalis*) on male flowers" by Ryotaro Kitajima, Osamu Matsuda, Koji Mastunaga, Ryotaro Hara, Atsushi Watanabe, Atsushi Kume to be considered for publication as an original research article in PLoS ONE.

In this manuscript, we evaluated the body temperature of the western conifer seed bug (WCSB, *Leptoglossus occidentalis*) on pine branches by field measurement and environmental biophysical analysis. WCSB is a pest of many pine species and is invasive worldwide and has recently been rapidly expanding its distribution in Japan. In early spring, we observed that most WCSBs gathered on male flowers rather than on female flowers and young cones. We hypothesized that male pine flowers were warmer than female flowers and needles, and WCSBs sucking male flowers may receive more heat. Our analysis supported this hypothesis. In addition, we found that the difference in color between the male and female pine flowers is a necessary condition for keeping the temperature of the male flowers at an appropriate temperature. As a result, it was also suggested that the reason why the colors of male and female flowers are different even though they are anemophiles is the result of temperature control of male flowers.

We suggest appropriate Academic Editors to handle our manuscript as follows: Abul Khayer Mohammad Golam Sarwar, Frank H. Koch, and Guillermo C. Amico.

We believe these findings will be of interest to the readers of your journal.

Thank you for your consideration.

Sincerely,

Atsushi Kume, PhD
Professor, Department of Agro-environmental Sciences
Faculty of Agriculture
Kyushu University

Manuscript
Click here to access/download;Manuscript;Leptoglossus_Kitajima3F.docx

Evaluation of thermoregulation of different pine organs in early spring and estimation of heat reward for the western conifer seed bug (*Leptoglossus occidentalis*) on male flowers

Short title: Heat reward estimation of western conifer seed bugs on male pine flowers

Ryotaro Kitajima[1], Osamu Matsuda[2], Koji Mastunaga[3], Ryotaro Hara[1], Atsushi Watanabe[1], Atsushi Kume[1]*

[1] Department of Agro-environmental Sciences, Faculty of Agriculture, Kyushu University, Motooka 744, Fukuoka, 819-0395, Japan

[2] Department of Biology, Faculty of Science, Kyushu University, Motooka 744, Fukuoka, 819-0395, Japan

[3] Kyushu Regional Breeding Office, Forest Tree Breeding Center, Forestry and Forest Product Research Institute, Forest Research and Management Organization, Suya 2320-5, Koshi, Kumamoto, 861-1102, Japan

* Corresponding author: akume@agr.kyushu-u.ac.jp

Abstract

The western conifer seed bug (WCSB, *Leptoglossus occidentalis*) is a pest of many pine species and is invasive worldwide. WCSB directly and indirectly deteriorates pine nut production by sucking seeds from cones. Currently, researchers think that WCSBs search for food by a combination of cues from visible light, infrared radiation, and chemicals such as monoterpenes. Some research revealed that WCSBs prefer larger cones, and it was thought that WCSBs suck seeds from and obtain more heat on larger cones. However, in early spring, we observed that most WCSBs gathered on male flowers rather than on female flowers and young cones. We hypothesized that male pine flowers were warmer than female flowers and needles, and WCSBs sucking male flowers may receive more heat. To test these hypotheses, we measured spectral reflectance with a hyperspectral sensor and temperature of pine organs with tiny thermocouples, and the data were analyzed by a heat budget model. Our results revealed that male flowers were significantly warmer and more reflective than female flowers and needles, which may attract WCSBs. These results supported our hypothesis that WCSBs on male flowers were warmer than those on other organs. This study will help further understanding of WCSBs and the adaptive value of pine flower colors.

Introduction

The western conifer seed bug (WCSB, *Leptoglossus occidentalis*) is native to North America and a pest that affects pine nut production by sucking seeds from cones [1–4]. WCSBs are spreading worldwide [5,6]; therefore, development of methods to control WCSBs is needed [7]. WCSBs can overwinter in litter or buildings, and appear in spring

[8]. These overwintering WCSBs suck developing male flowers and make them stunted [8]. It is also known that WCSBs prefer pine cones. Cone preference is not related to tree height or density of seed cones, but to the size of seed cones, with preference for larger cones [9,10]. Additionally, a monoterpene profile difference was detected between preferred and non-preferred seed cones [9]. Furthermore, the WCSB has an organ to detect infrared radiation and is attracted by infrared radiation [11]. Although ~~there are~~ several studies ~~that~~ proposed search cues used by WCSBs, the effectiveness of each does not seem to be definitive. Currently, it is thought that WCSBs search for seed cones by a combination of shortwave light, infrared radiation, and chemical cues [9].

The body temperature of insects is important because they are poikilotherm and strongly affected by external conditions. If the temperature is low, their activity is limited and they cannot fly. For example, bumblebees (*Bombus*) can fly in cold temperatures in early spring by warming up their thoracic temperature [12,13]. Similar phenomena have been investigated in dragonflies, whose optimal thoracic temperature is approximately 40 °C [14]. Cicadas, which belong to the same order as *L. occidentalis*, regulate their body temperature by metabolic generation and solar radiation [15]. Although it is not known whether WCSBs have an optimal temperature, it is possible that early spring is cold for WCSBs.

Pinus thunbergii belongs to Pinaceae, and all *Pinus* species are anemophilous. Because the flowers are wind-pollinated, there is no need to attract pollinators with flower color. However, *P. thunbergii* exhibits different colors during early spring flowering based on anthocyanin content [16], with yellow male flowers and red female flowers. Although the reason why female flowers are red has been discussed [17–19], the actual cause is still unclear. In addition, no studies have focused on the color difference between male and

female flowers. ~~Therefore, it has not been revealed why male and female flowers have different colors.~~

A heat budget (energy balance) model can physically estimate the temperature of an object and be used for living organisms [20]. For small objects such as WCSBs, heat storage can be ignored; therefore, WCSB input and output energy can be considered balanced. Such models can be applied to insects and small plant organs to evaluate the effects of color and morphology [20].

At a pine tree garden in Kumamoto Prefecture, Japan, most adult WCSBs were observed on developing male flowers in early spring. Because WCSBs can detect infrared radiation, we hypothesized that male flowers were warmer than needles and female flowers (Fig 1). Furthermore, WCSBs may receive heat rewards from male flowers. Therefore, we investigated ~~and discussed~~ the difference in temperature and spectral reflectance among pine organs relative to their thermoregulation, and estimated the difference in temperature between WCSBs on different pine organs using a heat budget model. This study can provide insight that may have biological application; for example, pine flower thermoregulation and an effective heat budget model may be useful for controlling WCSBs.

Materials and Methods

Study site and plant materials

We conducted our study at the experimental crossing garden of Forest Tree Breeding Center Kyushu Regional Breeding Office (Kumamoto, Japan, 32.8816°N, 130.7357°E) on 1 April 2021, and the roof of West 5 Building of Kyushu University (Fukuoka, Japan,

33.5942°N, 130.2146°E) on 19 January 2021. In Kumamoto, *P. thunbergii* grafted in 2015 was in bloom on 1 April. The trees were pruned to a height of approximately 3 m in late fall. Male flowers, female flowers, and needle leaves were sampled, and the spectral reflectance was measured the next day.

Spectral reflectance measurement

Spectral reflectance was measured by a pushbroom scanning system. The imaging system was similar to those in previous studies [21,22] except that the light source consisted of a pair of DC halogen lamps. A line-scanning hyperspectral camera (VNIR-200R, Themis Vision Systems, Bay St Louis, USA) sensitive to visible to near-infrared wavelengths (450–980 nm) was used. The camera was equipped with a silicon charge-coupled-device detector and was capable of acquiring 1,392 × 1,040 pixel images with 12-bit digitization and at 1.3-nm spectral resolution. The reflectance was obtained based on a white filter paper placed beside the stage as a reference surface. The visible (450–780 nm) and near-infrared (780–980 nm) reflectance of the filter paper were 93.0% and 97.8%, respectively, and the obtained reflectance was calibrated with a PTFE standard reflector (SphereOptics GmbH, Uhldingen, Germany). After capturing the images, the area of the WCSB was manually extracted from each image. The spectral reflectance of each WCSB was determined by averaging the spectral reflectance of each pixel.

Measurements

To establish heat budget model, environmental conditions were measured on sunny day, 1 April 2021. Air temperature and humidity were measured by Thermo Recorder (TR-72wb, T&D Co., Tokyo, Japan) at 1.9 m above ground. Shortwave radiation was measured by a pyranometer (SP-110-SS, Apogee Instruments, Inc., Logan, USA). Temperatures of male flowers, female flowers, and needles in the same shoots were

measured with thermocouples (ϕ=0.12 mm). Sample sizes were three for male and female flowers and two for needles. Wind speed and ground temperature were estimated from micrometeorological measurements. The obtained organ temperatures were used for further accuracy test.

WCSB body temperature was measured on 19 January 2022 on the roof of Kyushu University West 5 Building using dead WCSBs. The thermocouples were attached to each dead WCSB body with cyanoacrylate adhesive, and to a polyvinyl chloride (PVC) tube with tape. The detailed size and structure are shown in Fig 2.

WCSB orientation was set to horizontal and vertical. The PVC tube was used to simulate the effect of objects near the WCSB and its shortwave reflectance was set to 0.2. Air temperature and humidity were measured by Thermo Recorder. Wind speed was measured using a sonic anemometer (ATMOS-22, METER Group, Inc., Pullman, USA) at 1.2 m above ground. All data were recorded once per 30 s and averaged over 10 min. Heat budget model accuracy was tested by root mean square error (RMSE), bias, and determination coefficients.

The heat budget model also required shortwave absorptance. This was obtained by integration of spectral reflectance and spectral radiation. Because spectral characteristics differ between sunny and cloudy days [23], absorption differs depending on the weather. Spectral radiation was measured on the roof of West 5 Building of Kyushu University (Fukuoka, Japan) with an MS-711 spectroradiometer (EKO Instruments Co., Ltd., Tokyo, Japan) with a rotating shadow band (RSB-01, EKO Instruments Co., Ltd., Tokyo, Japan). Because it was sunny on 1 April 2021, spectral radiation on a sunny day was used to calculate absorptance.

Heat budget formulae

We made a heat budget model from the following formulae [20]. We simulated the surface temperature of WCSBs on a needle, a male flower inflorescence, and a female flower(Fig 3). To calculate heat budget, the WCSB was approximated as an ellipsoid whose long and short axes were 15 mm and 5 mm, respectively, and its leg length was set to 5 mm. The female flower was also approximated as an ellipsoid, whose long and short axes were 11 mm and 8 mm, respectively. The needle was approximated as a pillar whose radius and length were 1 mm and 100 mm, respectively. Male flower inflorescence was approximated as a pillar whose radius and height were set to 11 mm and 45 mm, respectively.

Radiation input was obtained from the ~~below~~ formulae.

$$R_{in} = R_{sd}(1-\alpha_p)\left(p_{dir}\, F_d + p_{diff}\left(F_{sky} + a_{object}\, F_{object} + a_{ground}\, F_{ground}\right)\right) + \left(F_{sky}\, R_{ld} + F_{ground}\, R_{lu} + F_{object}\, R_{lobject}\right) \quad (1)$$

$$R_{ld} = \sigma T_g^4 \quad (2)$$

$$R_{lu} = \sigma(T_a - 20)^4 \quad (3)$$

$$R_{lobject} = \sigma T_{object}^4 \quad (4)$$

where R_{in} means the total radiation input (W m^{-2}), R_{sd} is shortwave radiation, α_p is reflectance obtained from the integration of spectral reflectance and spectral radiation (W m^{-2}), and p_{dir} and p_{diff} represent the ratio of direct radiation and diffuse radiation in total radiation [24], respectively. α_{ground} and α_{object} are shortwave reflectance of the ground and object, respectively. R_{ld}, R_{lu}, and $R_{lobject}$ are longwave radiation from the sky, ground, and object (W m^{-2}), respectively. F_d, F_{ground}, and F_{object} are view factors of the sky, ground, and object, respectively. σ is Stefan–Boltzmann constant. T_g and T_a are ground and air

temperature (K), longwave radiation of the sky was estimated from assuming sky as a blackbody cooler than atmosphere by 20 K [25].

$$R_{net} = R_{in} - \sigma T_s^4 \quad (5)$$

R_{net} is net radiation obtained from subtracting longwave radiation from input radiation. T_s is the temperature of the target object.

$$R_{net} = H + lE \quad (6)$$

$$H = c_p \times 1.4 \times 0.135 \times \sqrt{\frac{u}{d}}(T_s - T_a) \quad (7)$$

$$lE = l \times 1.4 \times 0.147 \times \sqrt{\frac{u}{d}}\frac{e(T_s) - q_a}{\mathrm{P_a}} \quad (8)$$

where H is a sensible heat exchange (H: W m^{-2}), c_p is specific heat at constant pressure of atmosphere (J mol^{-1} K^{-1}), u is wind speed (m s^{-1}), d is the characteristic length (m), lE means latent heat exchange (lE: W m^{-2}), l is latent heat of vaporization (kJ mol^{-1}), and E is water vapor flux density (mol m^{-2}). e(T_s) is saturated vapor pressure at T_s, q_a is water vapor pressure, and P_a is atmospheric pressure. In this study, the heat storage and latent heat exchange from the WCSB surface were ignored; therefore, R_{net} was dispersed only by sensible heat exchange (H). For *P. thunbergii* organ temperature, R_{net} was dispersed by both latent heat and sensible heat.

Determination of view factor

F_d represents the view factor of objects for direct radiation and was calculated from below formulae (9) for vertical, (10) for horizontal and (11) for shape ratio,

$$F_d = \frac{\sqrt{1 + (x^2 - 1)\sin\theta}}{2x + \frac{\left(2\sin^{-1}\sqrt{1 - x^2}\right)}{\sqrt{1 - x^2}}} \quad (9)$$

$$F_d = \frac{\sqrt{1 + (x^2 - 1)\cos\theta}}{2x + \frac{\left(2\sin^{-1}\sqrt{1 - x^2}\right)}{\sqrt{1 - x^2}}} \quad (10)$$

$$x = \frac{a}{b} \quad (11)$$

where θ represents solar elevation, and a and b represent the long and short axes of ellipsoids, respectively.

Other view factors (F_{sky}, F_{ground}, and F_{object}) were different depending on kind of objects and distances from the objects. They were estimated using TouchDesigner 2020.25380 (Derivative Inc., Toronto, Canada), a 3D modeling software program, and ImageJ 1.53K [26].

We prepared “Tube SOP” as the needle and male inflorescence and “Sphere SOP” as the female flower in TouchDesigner. “Camera COMP” was placed 5 mm away from the objects. To obtain the scene from Camera COMP, “Render TOP” was used and set to “Fish-Eye (180)” to obtain 180 FOV pictures. Obtained pictures were output by “Movie File Out TOP” and input in ImageJ. The area ratio of each object (sky, ground, and objects) was obtained by ImageJ’s threshold feature.

In view factors for *P. thunbergii*, F_{object} was set to 0, and F_{sky} and F_{ground} were set to 0.5. F_d was obtained from formula (9).

Statistical analysis

A time-based analysis ~~that~~ was conducted along with analysis of variance (ANOVA) ~~was conducted~~ in R 4.0.2 [27]. Heat budget calculation was conducted in R 4.0.2 [27] with the packages “suncalc” [28] and “nleqslv” [29].

Results

Spectral reflectance

Fig 4 shows the spectral reflectance of *P. thunbergii* organs and *L. occidentalis*. Among

the three *P. thunbergii* organs, the male flower had the highest reflectance. Female flowers and needles had low reflectance but relatively high reflectance in the red range for female flowers and in the green range for needles. *Leptoglossus occidentalis* had low reflectance over the measured wavelength range and relatively high reflectance in long wavelengths.

Field measurements

Figs 5 and 6 show the measurements recorded at Kyushu University on 19 January 2022. The results showed that WCSBs on PVC tube were warmer than other WCSBs. WCSBs on PVC tube were warmer than the air temperature by approximately 5 °C. These values were further used for the heat budget model and accuracy test.

Figs 7 and 8 show the environmental conditions and organ temperature measurements in the field on 1 April 2021 at the tree garden. Male flower temperature was the highest of the three organs, and the temperatures of the other two organs were similar to each other. The ANOVA results showed that there was a significant difference between male and female flower temperatures. The environmental conditions were used for heat budget calculation.

View factors

Fig 9 shows the views from WCSBs on different objects, and Table 1 shows the obtained view factors from Fig 9. Excluding male flowers and PVC pipe, the ~~upside~~ view factors were dominated by sky. On male flowers, the area occupied by the male flower was approximately one-third of the area. Thus, view factors were influenced by object size. These values were used for heat budget calculations.

Table 1. Detailed values of view factors (F) for heat budget calculation. For example, F_{sky} for WCSBs on male flowers can be seen under the "Male" row and "Sky" column.

		Sky	Ground	Object
Female	Upward	0.500	0.000	0.000
	Downward	0.013	0.415	0.072
Male	Upward	0.347	0.000	0.153
	Downward	0.009	0.344	0.147
Needle	Upward	0.500	0.000	0.000
	Downward	0.013	0.466	0.021
Tube	Upward	0.341	0.000	0.159
	Downward	0.009	0.328	0.162

Estimation of male and female flower temperatures

Temperatures of male and female flowers were estimated with accuracies of 2.04 °C and 0.41 °C in RMSE and bias, respectively. To confirm the effect of different colors, we estimated the temperature using objects with female flower color and male flower size, and male flower color with female flower size under the microclimate conditions recorded on 1 April 2021. The temperature of the object with female flower color and male flower size was 29.0 °C on average (31.0 °C maximum). These values were higher than those of the estimated male flower temperature by 0.7 °C on average (0.9 °C maximum). The temperature of the object with male flower color and female flower size was 27.4 °C on

average (28.8 °C maximum). These values were lower than those of the estimated female flower temperature by 0.4 °C on average (0.5 °C maximum).

Estimation of *L. occidentalis* temperature

Fig 10 shows the accuracy of the model estimating WCSB temperature. RMSE, bias, and determination coefficient were 1.52 °C, –0.39 °C, and 0.341, respectively. However, the WCSB temperature near the PVC tube was underestimated, and RMSE, bias, and determination coefficient were 2.26 °C, –2.12 °C, and 0.559, respectively.

Under the environmental conditions on 1 April 2021, WCSBs on male flowers were warmer than those on the other two organs (Fig 11). The mean temperature difference was approximately 1 °C during this period. The temperatures of WCSBs on needles and female flowers were approximately the same.

Discussion

Clarifying WCSB host tree preference is important for controlling WCSB populations. Because of their importance, previous researchers tried to identify and hypothesize visible light cues [10], infrared radiation cues [11], and volatile chemical compound cues [9]. However, some researchers could not find differences in certain cues, such as infrared radiation from cones [9]. WCSB host preference has puzzled many researchers and is still unclear [9–11,30]. The present study provides insight based on the thermoregulation strategy of pine and micrometeorology.

There is a potential trade-off between thermoregulation and maximizing offspring production in pine male flowers, and male flower color may be adaptive. Because Pinaceae species are anemophilous, the color of flowers would not need to attract pollinators. In female flowers, this has been discussed in the context of three hypotheses:

thermoregulation, photoprotection, and anti-herbivory [17–19,31]. However, there are no studies that focused on male flower color and its adaptive implications. Pine trees need to produce a lot of pollen and male flowers [32]. Large male inflorescences are attached, and large and dense inflorescences are inefficient in exchanging heat with air (Eqs. (7) and (8)). Although pine pollen is resistant to temperature and dryness once released, male flowering is considered sensitive to high temperatures, and over 30 °C is potentially harmful [33,34]. Therefore, the higher reflectance in male flowers may indicate a ~~heat~~ heat-blocking strategy to reduce the amount of radiation input. The pigmentation with anthocyanins may be advantageous even in male flowers because the female red color is thought to play some protection roles, especially against UV radiation. However, an object with female flower color and male flower size was estimated to exceed 30 °C; therefore, the simultaneous pigmentation protection may cause heat stress and be lethal for male flowers. Therefore, such protection by anthocyanins could be a “privilege” only for female flower because of its high cooling efficiency, and male flowers could be forced to have high reflectance to not get too warm.

In early spring, WCSB are more commonly found on male flowers. Male flowers have higher reflectance for shortwave radiation (Fig 4) and higher temperatures (Fig 7). Objects with high temperature emit more infrared radiation, and WCSBs may be able to detect it. In addition, higher temperatures result in greater release of volatile compounds such as monoterpenes. Therefore, male flowers may be more conspicuous to WCSBs because of their high reflectance, high temperature, and high concentration of volatile compounds.

WCSBs suck and get heat from male flowers. WCSBs on male flowers were warmer than individuals on needles or female flowers, and the mean temperature difference was

estimated to be at least 1 °C. Here, we emphasize that this model does not consider the differences in the characteristic length of organs. Therefore, the difference in temperature was only caused by difference in radiation balance. However, Fig 10 shows that there was a gap between measured and estimated air temperatures that cannot be explained by the radiation balance. Large objects obstruct the wind, which results in less heat exchange. The gap in Fig 10 can be explained by this hindered heat exchange. Fig 10 shows that WCSBs on male flowers can be 3 °C warmer than individuals on needles and female flowers.

Conclusion

This study quantified the body temperature of WCSBs on different pine organs and addressed pine flower thermoregulation. A heat budget model based on field measurements was applied to estimate temperatures of WCSBs and pine organs. Field measurements showed that male flowers were significantly warmer and more reflective than other organs, which indicates that some features of male flowers may attract WCSBs. Additionally, flower colors may represent a thermoregulation strategy. The heat budget model showed that the temperatures of WCSBs on male flowers on a clear day were more than 1 °C higher than those of the individuals on female flowers and needles. Because this model only considered the effect of the radiation balance and did not include the effects of boundary layer resistance, the actual effect of the temperature increase on male flowers would be approximately 3 °C. This result emphasizes the advantage of staying on male flowers in early spring because WCSBs can both suck the male flowers and receive heat. This study provides insight into WCSB host selection and methods for estimating the temperature environment of insects.

Acknowledgments

This study was partly supported by JSPS KAKENHI Grant Number JP18H02511. We thank Mallory Eckstut, PhD, from Edanz (https://jp.edanz.com/ac) for editing a draft of this manuscript.

References

1. Heidemann O. New species of *Leptoglossus* from North America. Proc Entomol Soc Washingt. 1910.
2. Farinha AO, Carvalho C, Correia AC, Branco M. Impact assessment of *Leptoglossus occidentalis* in *Pinus pinea*: Integrating population density and seed loss. For Ecol Manage. 2021;496: 119422. doi:10.1016/J.FORECO.2021.119422.
3. El Khoury Y, Noujeim E, Bubici G, Tarasco E, Al Khoury C, Nemer N. Potential Factors behind the Decline of *Pinus pinea* Nut Production in Mediterranean Pine Forests. Forests. 2021. doi:10.3390/f12091167.
4. Schowalter TD, Sexton JM. Effect of *Leptoglossus occidentalis* (Heteroptera: Coreidae) on Seed Development of Douglas-Fir at Different Times During the Growing Season in Western Oregon. J Econ Entomol. 1990;83: 1485–1486. doi:10.1093/jee/83.4.1485.
5. Barta M. Biology and temperature requirements of the invasive seed bug *Leptoglossus occidentalis* (Heteroptera: Coreidae) in Europe. J Pest Sci (2004). 89. doi:10.1007/s10340-015-0673-z.
6. Byeon DH, Jung JM, Jung S, Lee WH. Distribution analysis of *Leptoglossus occidentalis* Heidemann (Heteroptera: Coreidae) in South Korea using climate

and host plant ensemble maps. J Asia Pac Entomol. 2021;24: 1077–1086. doi:10.1016/j.aspen.2021.10.003.

7. Lesieur V, Farinha AO. Responses of native egg parasitoids to the invasive seed bug *Leptoglossus occidentalis*. Agric For Entomol. 2021;23: 323–333. doi:10.1111/afe.12434.

8. Koerber TW. *Leptoglossus occidentalis* (Hemiptera, Coreidae), a Newly Discovered Pest of Coniferous Seed. Ann Entomol Soc Am. 1963;56: 229–234. doi:10.1093/aesa/56.2.229.

9. Richardson TA, Strong WB, Aukema BH, Takàcs S, Zahradnik T, Lindgren BS. Assessment of cues potentially mediating host selection of *Leptoglossus occidentalis* on *Pinus contorta*. Agric For Entomol. 2017;19: 245–256. doi:10.1111/afe.12203.

10. Blatt SE, Borden JH. Physical characteristics as potential host selection cues for *Leptoglossus occidentalis* (Heteroptera : Coreidae). Environ Entomol. 1999;28: 246–254. doi:10.1093/ee/28.2.246.

11. Takács S, Bottomley H, Andreller I, Zaradnik T, Schwarz J, Bennett R, et al. Infrared radiation from hot cones on cool conifers attracts seed-feeding insects. Proc R Soc B Biol Sci. 2009;276: 649–655. doi:10.1098/rspb.2008.0742.

12. Bernd H. Temperature Regulation in the Bumblebee *Bombus vagans*: A Field Study. Science (80-). 1972;175: 185–187. doi:10.1126/science.175.4018.185.

13. Heinrich B, Kammer AE. Activation of fibrillar muscles in bumblebee during warm-up, stabilization of thoracic temperature and flight. J Exp Biol. 1973;58: 677–688.

14. Marden JH, Kramer MG, Frisch J. Age-related variation in body temperature,

thermoregulation and activity in a thermally polymorphic dragonfly. J Exp Biol. 1996;199: 529–535.

15. Sanborn AF. Comparative thermoregulation of sympatric endothermic and ectothermic cicadas (Homoptera : Cicadidae : Tibicen winnemanna and Tibicen chloromerus). J Comp Physiol A-SENSORY NEURAL Behav Physiol. 2000;186: 551–556. doi:10.1007/s003590000110.

16. Griesbach RJ, Santamour FS. Anthocyanins in cones of *Abies*, *Picea*, *Pinus*, *Pseudotsuga* and *Tsuga* (*Pinaceae*). Biochem Syst Ecol. 2003;31: 261–268. doi:10.1016/S0305-1978(02)00147-3.

17. Rudall PJ. Colourful cones: how did flower colour first evolve? J Exp Bot. 2020;71: 759–767. doi:10.1093/jxb/erz479.

18. Sturgeon KB, Mitton JB. Cone color polymorphism associated with elevation in white fir, *Abies concolor*, in southern colorado. Am J Bot. 1980;67: 1040–1045. doi:10.1002/j.1537-2197.1980.tb07735.x.

19. Geburek T, Robitschek K, Milasowszky N, Schadauer K. Different cone colours pay off: lessons learnt from European larch (*Larix decidua*) and Norway spruce (*Picea abies*). Can J Bot. 2007;85: 132–140. doi:10.1139/B07-003.

20. Campbell GS, Norman JM. An Introduction to Environmental Biophysics. 2nd ed. New York: Springer-Verlag; 1998.

21. Matsuda O, Tanaka A, Fujita T, Iba K. Hyperspectral imaging techniques for rapid identification of arabidopsis mutants with altered leaf pigment status. Plant Cell Physiol. 2012;53: 1154–1170. doi:10.1093/pcp/pcs043.

22. Wakabayashi H, Matsuda O, Fujita T, Kume A. Practical application of proximal sensing for monitoring the growth of *Physcomitrium patens*. Biol Sci Sp.

2021;35: 32–40. doi:10.2187/bss.35.32.

23. Kume A, Akitsu T, Nasahara KN. Why is chlorophyll b only used in light-harvesting systems? J Plant Res. 2018;131: 961–972. doi:10.1007/s10265-018-1052-7.

24. Liu BYH, Jordan RC. The interrelationship and characteristic distribution of direct, diffuse and total solar radiation. Sol Energy. 1960;4: 1–19. doi:https://doi.org/10.1016/0038-092X(60)90062-1.

25. Jones HG. Plants and Microclimate: A Quantitative Approach to Environmental Plant Physiology, 3rd ed. Cambridge University Press; 2013.

26. Schneider, C. A., Rasband, W. S., Eliceiri KW. NIH Image to ImageJ: 25 years of image analysis. Nat Methods. 2012;9: 671–675.

27. R Core Team. R: A Language and Environment for Statistical Computing. Vienna, Austria; 2020. Available from: https://www.r-project.org/

28. Benoit T, Elmarhraoui A. suncalc: Compute Sun Position, Sunlight Phases, Moon Position and Lunar Phase. 2019. Available from: https://cran.r-project.org/package=suncalc

29. Hasselman B. nleqslv: Solve Systems of Nonlinear Equations. 2018. Available from: https://cran.r-project.org/package=nleqslv

30. Farinha AO, Durpoix C, Valente S, Sousa E, Roques A, Branco M. The stone pine, *Pinus pinea* L., a new highly rewarding host for the invasive *Leptoglossus occidentalis*. NEOBIOTA. 2018; 1–18. doi:10.3897/neobiota.41.30041.

31. Zahradnik T, Takács S, Strong W, Bennett R, Kuzmin A, Gries G. Douglas-fir cone gall midges respond to shape and infrared wavelength attributes of host tree branches. Can Entomol. 2012/08/21. 2012;144: 658–666.

doi:10.4039/tce.2012.71.

32. Cruden RW. Pollen grains: why so many? PLANT Syst Evol. 2000;222: 143–165. doi:10.1007/BF00984100.

33. Flores-Rentería L, Whipple A V, Benally GJ, Patterson A, Canyon B, Gehring CA. Higher Temperature at lower elevation sites fails to promote acclimation or adaptation to heat stress during pollen germination. Frontiers in Plant Science. 2018. Available from: https://www.frontiersin.org/article/10.3389/fpls.2018.00536

34. Coescielny S. Effect of temperature, light and fungicides on the germination of pollen of some trees and shrubs. Rocz Dendrol Pol Tow Bot, Warsz,. 1962;23: 3178.

Figure captions

Fig 1. Western conifer seed bug (WCSB) holding on to male flowers. These pictures were taken on 8 April 2021.

Fig 2. Instruments for testing accuracy of the heat budget model. Thermocouples were attached to dead WCSBs and tips of 15-cm arms. A PVC tube was used to simulate the effect of obstacles near WCSBs.

Fig 3. Assumptions for heat budget estimation of WCSBs. Left: WCSB on a needle. Center: WCSB on male flowers. Right: WCSB on a female flower.

Fig 4. Spectral reflectance of pine plant organs and dead bodies of WCSB. Lines represent mean values.

Fig 5. Temperature measurements on the roof of West 5 Building of Kyushu University on 19 January 2022. Lines represent mean values and error bars represent standard deviation.

Fig 6. Environmental conditions at the roof of West 5 Building of Kyushu University on 19 January 2022. The top graph shows radiation and relative humidity, and the bottom graph shows wind speed and ground temperature.

Fig 7. Temperature measurements of *Pinus thunbergii*. Asterisks indicate a significant

difference between male and female flowers.

Fig 8. Environmental conditions in the field on April 1st, 2021. The top graph shows relative humidity and radiation measurements. The bottom graph shows the estimation result of wind speed at 1.9 m above ground and ground temperature. These results were used to calculate heat budget.

Fig 9. Views from WCSBs on needles, female flowers, male flowers, and PVC tubes.

Fig 10. Accuracy test of the heat budget model that estimated WCSB temperature. Root mean squire error, bias, and determination coefficient were 1.52 °C, –0.39 °C, and 0.341, respectively.

Fig 11. Estimated temperature of WCSBs on different plant organs. This estimation was conducted using environmental data from 1 April 2021.

Figure

Click here to access/download;Figure;Leptoglossus_Kitajima_Figures.docx

Figure 1

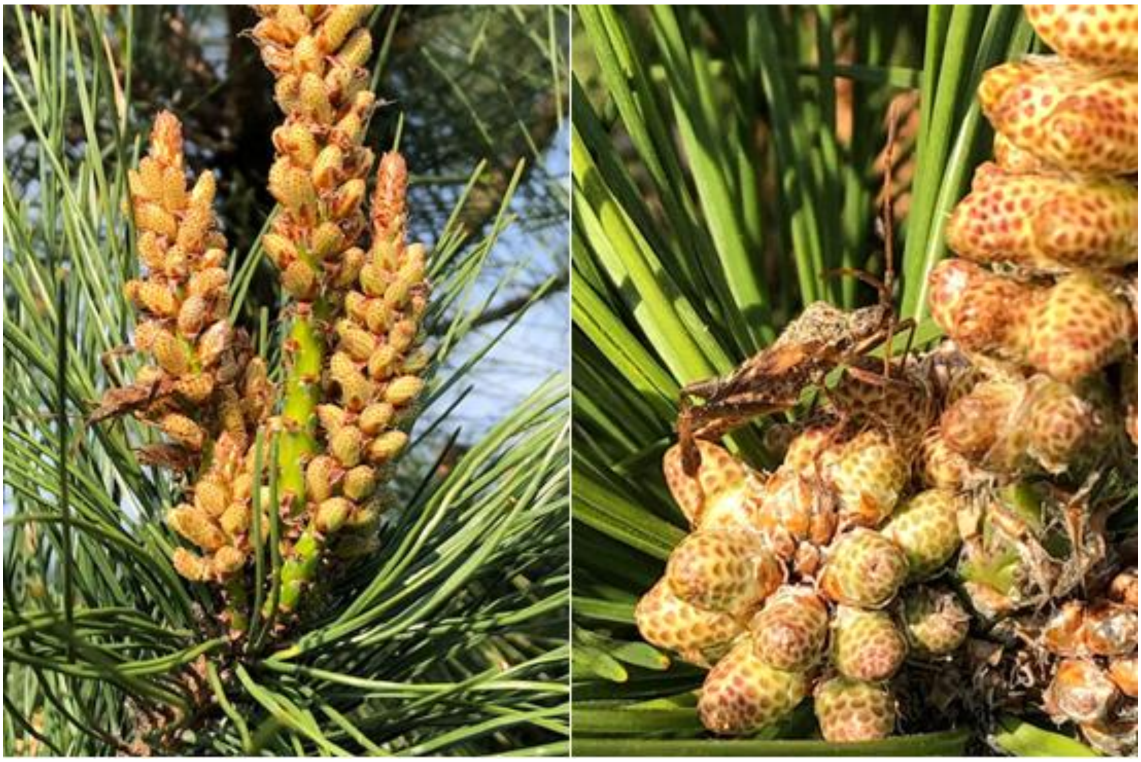

Figure 2

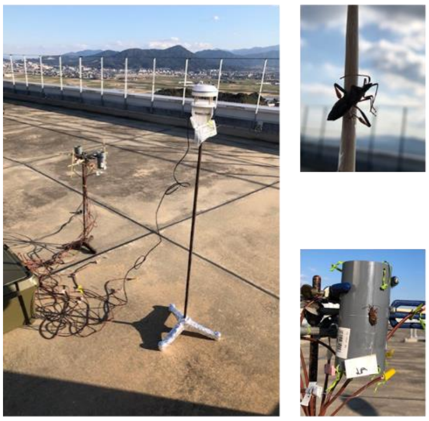

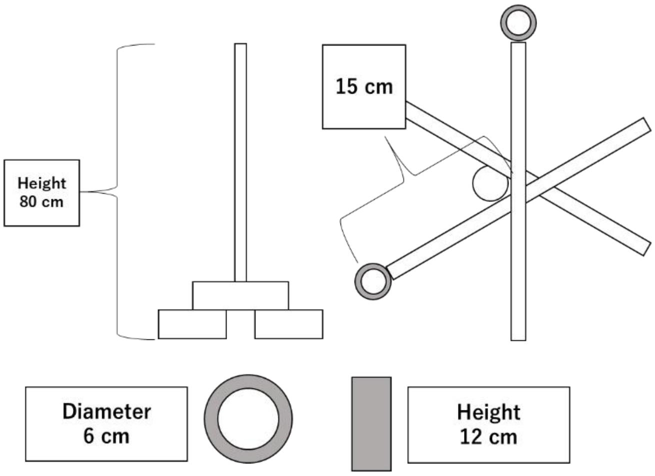

Figure 3

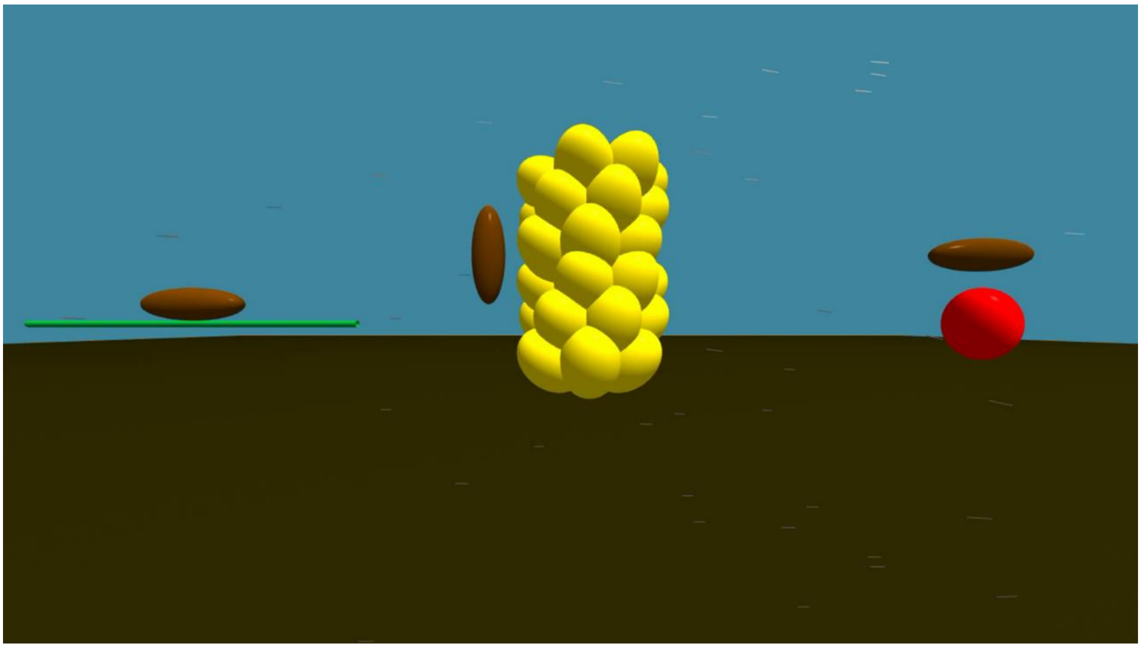

Figure 4

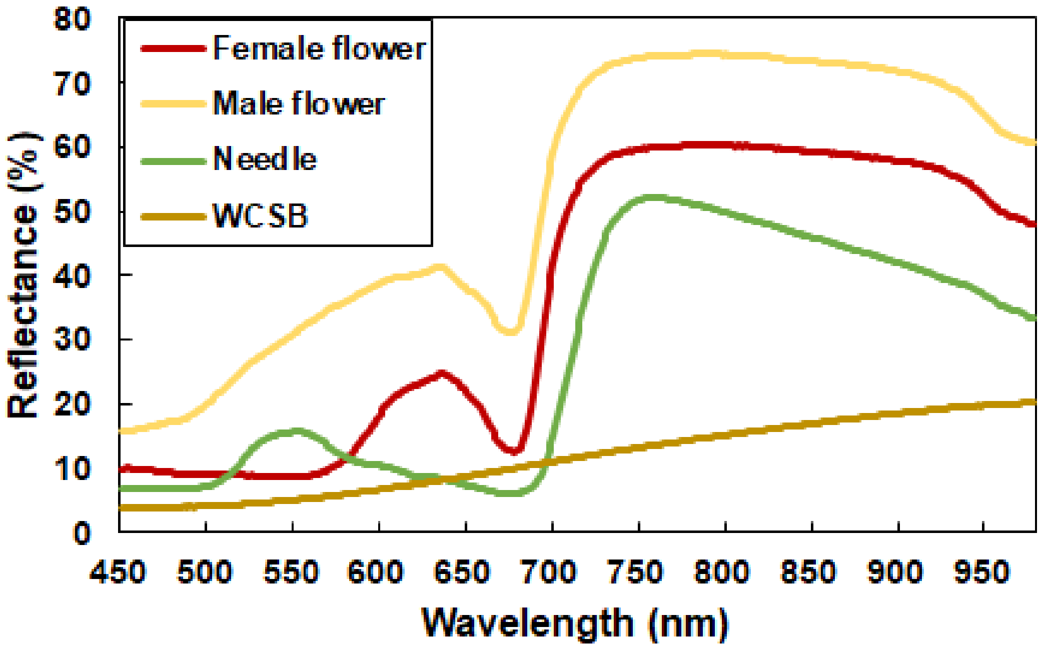

Female flower
Male flower
Needle
WCSB
Reflectance (%)
0
10
20
30
40
50
60
70
80
450
500
550
600
650
700
750
800
850
900
950
Wavelength (nm)

Figure 5

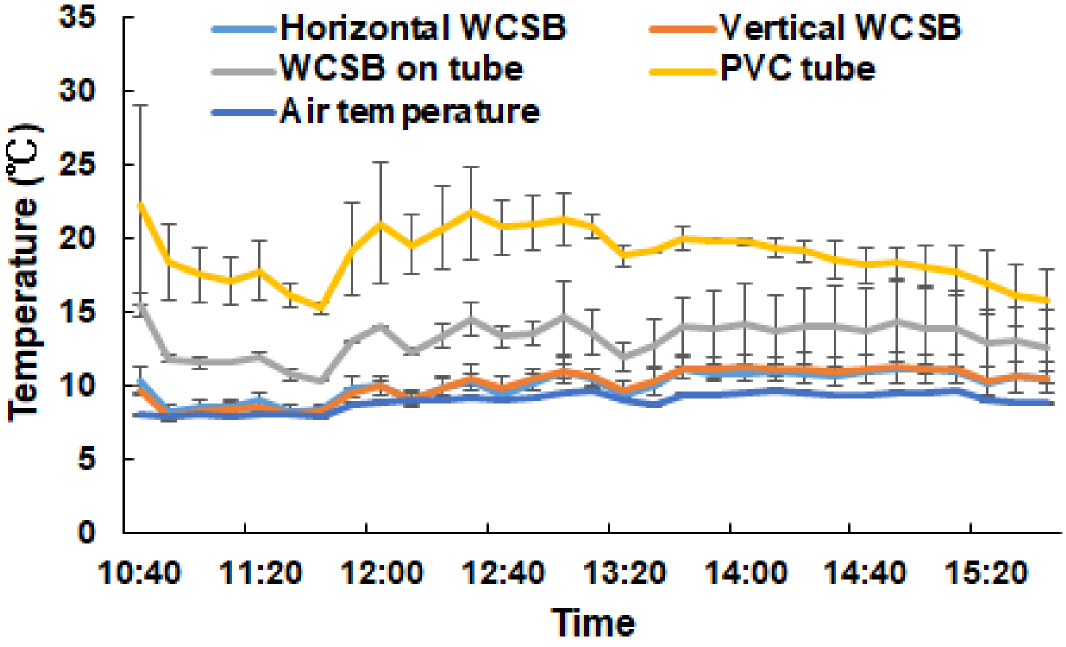
Horizontal WCSB
Vertical WCSB
WCSB on tube
PVC tube
Air temperature
Temperature (℃)
35
30
25
20
15
10
5
0
10:40
11:20
12:00
12:40
13:20
14:00
14:40
15:20
Time

Figure 6

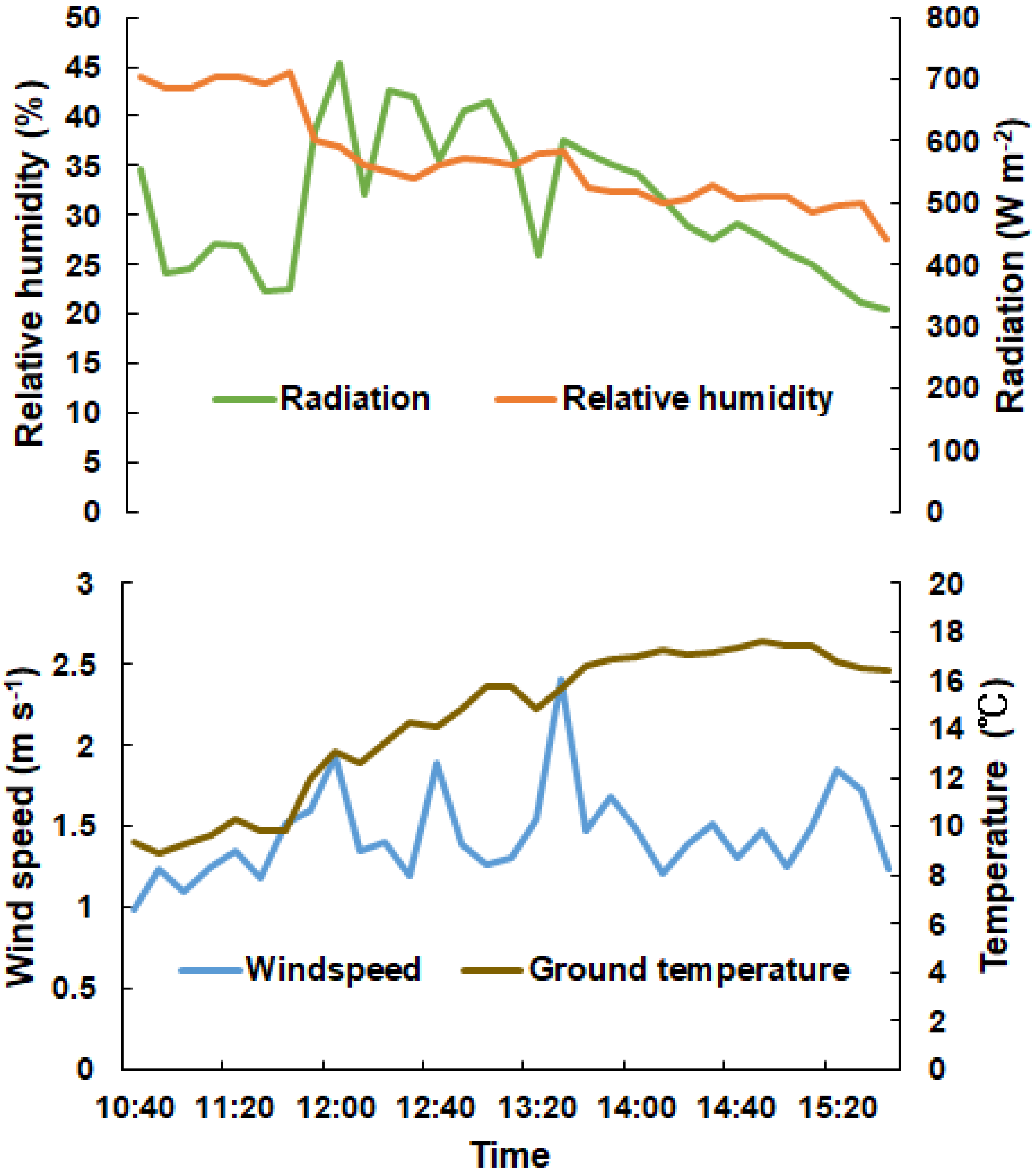

Relative humidity (%)
Radiation (W m-2)
Radiation
Relative humidity
Wind speed (m s-1)
Temperature (℃)
Windspeed
Ground temperature
10:40
11:20
12:00
12:40
13:20
14:00
14:40
15:20
Time

Figure 7

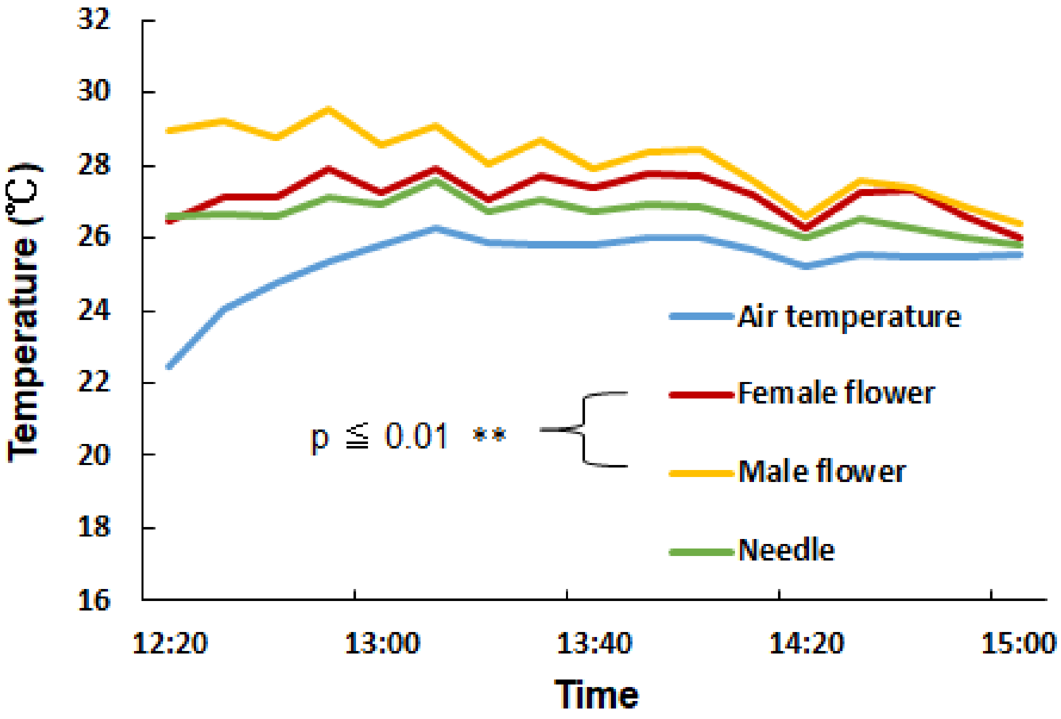

32
30
28
26
24
22
20
18
16
Temperature (℃)
12:20
13:00
13:40
14:20
15:00
Time
Air temperature
Female flower
Male flower
Needle
p ≦ 0.01 **

Figure 8

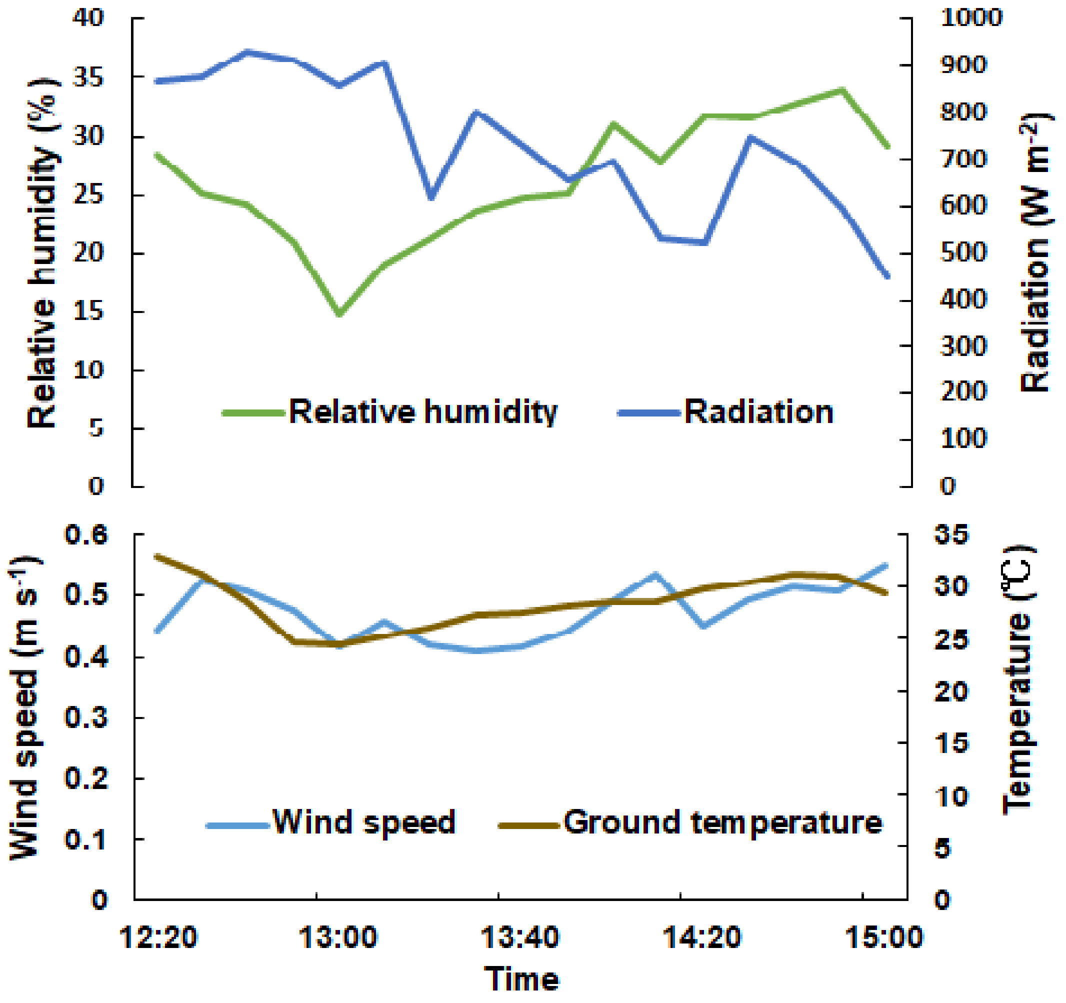

40
35
30
25
20
15
10
5
0
Relative humidity (%)
1000
900
800
700
600
500
400
300
200
100
0
Radiation (W m-2)
Relative humidity
Radiation
0.6
0.5
0.4
0.3
0.2
0.1
0
Wind speed (m s-1)
35
30
25
20
15
10
5
0
Temperature (℃)
Wind speed
Ground temperature
12:20
13:00
13:40
14:20
15:00
Time

Figure 9

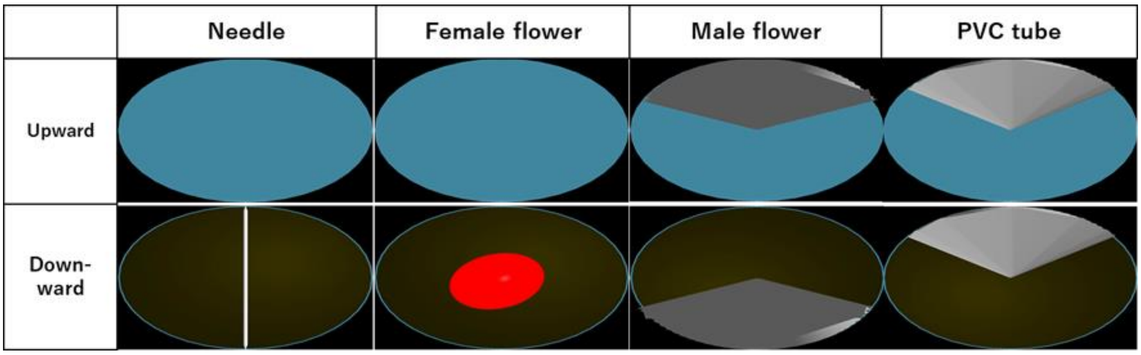

Figure 10

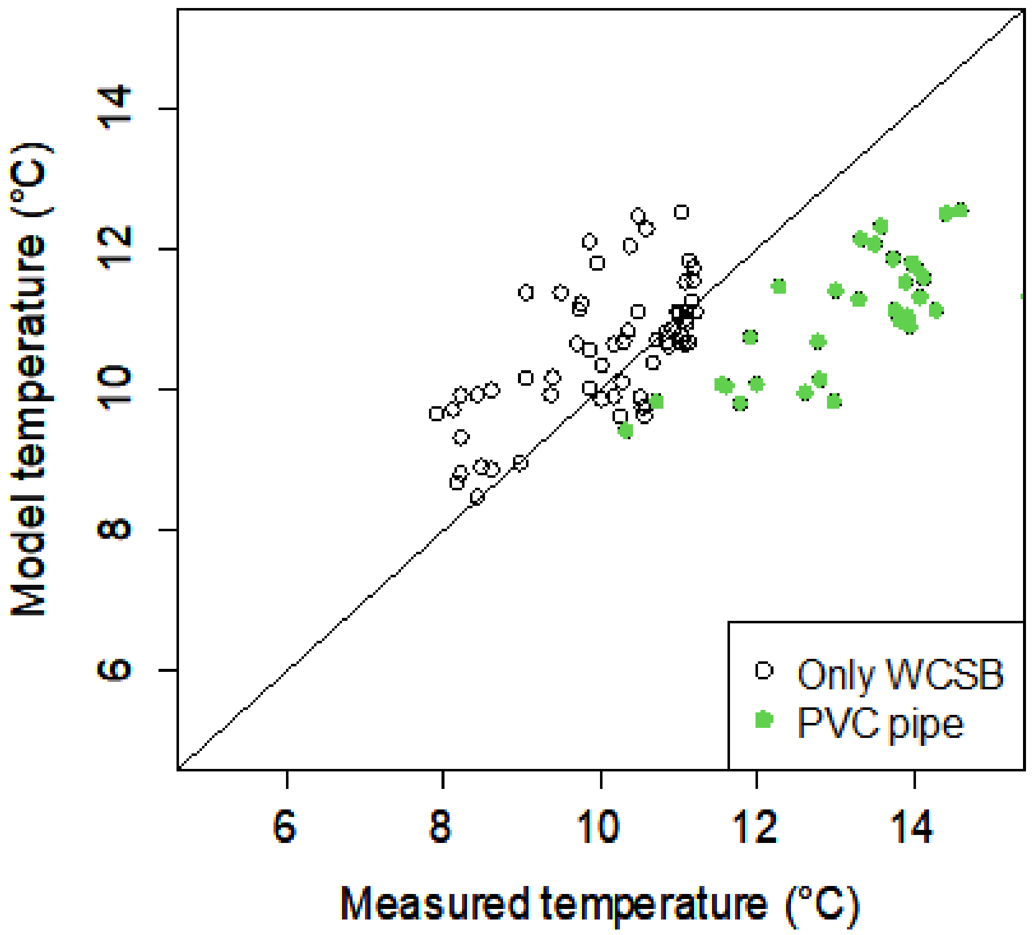

Model temperature (°C)
6
8
10
12
14
Measured temperature (°C)
6
8
10
12
14
Only WCSB
PVC pipe

Figure 11

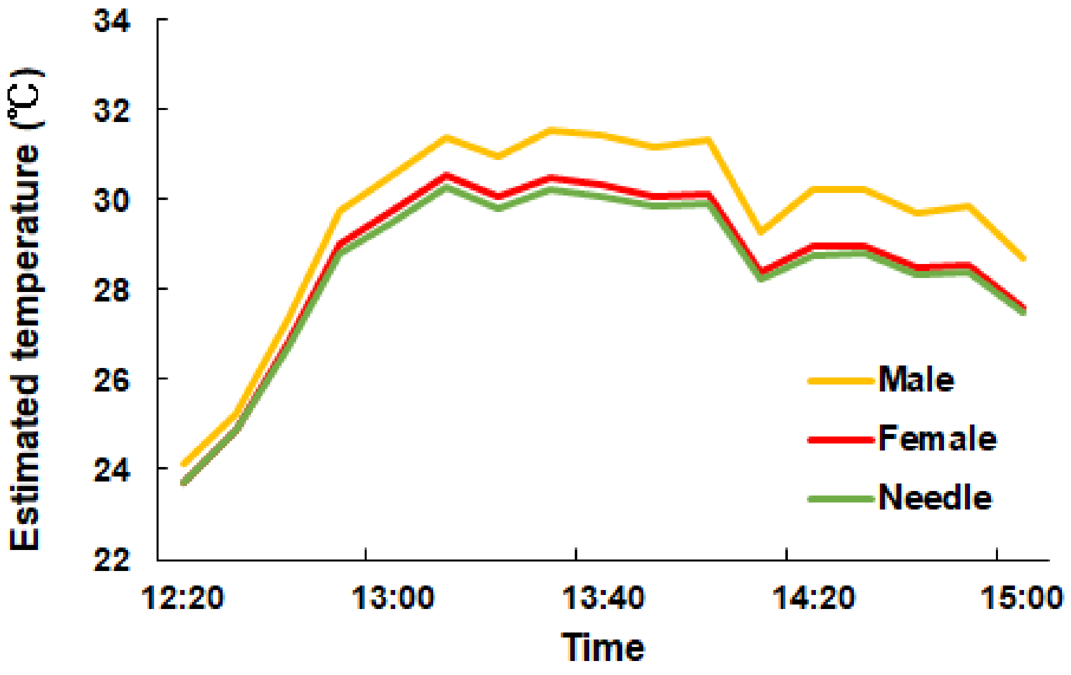

Estimated temperature (℃)
34
32
30
28
26
24
22
12:20
13:00
13:40
14:20
15:00
Time
Male
Female
Needle

Click here to access/download
Supporting Information - Compressed/ZIP File Archive
PLoS_Pthunbergii.ZIP
