# Supplementary material for: Evaluation of thermoregulation of different pine organs in early spring and estimation of heat reward for the western conifer seed bug (Leptoglossus occidentalis) on male cones
Source: PLoS One. 2022 Aug 4;17(8):e0272565. doi: 10.1371/journal.pone.0272565 (PMC9352051; doi:10.1371/journal.pone.0272565)
Supplement: Attachment — Submitted filename: PLOS_Response to Reviewers_1.docx [file pone.0272565.s006.docx]

# Response to Reviewers.

Dear Reviewers,

Thank you very much for the careful review of our submitted manuscript entitled " Evaluation of thermoregulation of different pine organs in early spring and estimation of heat reward for the western conifer seed bug (*Leptoglossus occidentalis*) on male flower ". Your feedback and the detailed comments have helped us to substantially improve the manuscript.

*Academic Editor*

*I have attached an annotated version of your manuscript PDF with some editorial suggestions. Although I recognize that some other researchers have called immature pine cones "flowers", I discourage you from using this terminology since pines (as gymnosperms) are not flowering plants. It could be confusing to some readers. If you choose to keep the term flowers, you will need to define it for your audience.*

**Response 1:** Thank you for your kind suggestions. In particular, it was very helpful for us to correct the English expressions. The colors used in the figures have been completely changed. The expression of reproductive organs has been unified to "cones". As a result, the title was also changed. “Evaluation of thermoregulation of different pine organs in early spring and estimation of heat reward for the western conifer seed bug (*Leptoglossus occidentalis*) on male cones”

*Overall, I find myself in agreement with Reviewer 1: the conclusion that WCSB has a thermal preference for male cones/flowers is speculative. It could be true but there are other potential explanations for this preference that are just as plausible, including male cones/flowers having greater nutritional value or a more attractive suite of volatiles than female cones/flowers. Data on nutrients in male vs. female cones/flowers would be an excellent addition, but at the least, you should present and discuss some alternative explanations.*

**Response 2:** We fully agree with these opinions. In fact, there are many possibilities and we are still testing them. We have added additional information and discussions from lines 313 to 323.

*Reviewer #1: The manuscript presents original data which could explain a preference of WCSB for pine male flowers in early spring. Based on field observations indicating that WCSB prefers pine male flowers to feed, the authors estimated the temperature of male flowers, female flowers, and pine needles, to support the hypothesis of a thermal preference for this microhabitat. The experiments and analyses have a high technical standard and are well described. The article is well written.*

*The main drawback of the Discussion is that other alternative hypotheses for a preference for male flowers should be presented and discussed. In particular, male flowers’ nutritional value, such as sugars, and N content could be also more favorable for the bug. This could be discussed by presenting data on nutritional values or at least presenting this hypothesis. Also, volatiles released by male flowers and female flowers may be very dissimilar.*

**Response 3:** Thank you for evaluating our research. As mentioned in Response2, we have added a discussion about the alternative hypotheses.

*Other comments:*

*Line 48 – Add the information about this is the adult stage – adults WCSB*

**Response 4:** Thank you for the comment. I added such explanation in line 46.

*Line 110 – I did not follow this part - Do you refer to dead WCSB?*

**Response 5:** We added the explanation of the experimental condition in line 108.

*Lines 121-124 – How many bugs were tested for each treatment?*

**Response 6:** Thank you for the question. The sample size was two and the sentence was added in line 131.

*Lines 312-313 – I found this part of the conclusion speculative. The authors do not have enough information to say the bugs receive significant heat just by sucking on male flowers rather than on female flowers. The results provided by the authors mainly demonstrate that by staying in male flowers the bugs may receive more heat from this microhabitat which may elevate their body temperature above air temperature. What is ingested and the respective energy budget, including the metabolic heat produced, are completely out of the scope of this work. In fact, should be discussed whether the nutritional value and sugar content of male flowers and female flowers also differ.*

**Response 7:** We fully agree with these suggestions. We changed the conclusion and added description about other hypotheses in discussion from lines 313 to 323.

*Reviewer #2: In general a simple but nice paper providing data on a worldwide pest. It can be improved better stating the objectives of the work, improving introduction in terms of invasiveness of WCSB and economic damage.*

**Response 8:** Thank you very much for the comment. I added the damage information in introduction from lines 42 to 44.

*Also I`d like to see discuss a bit on overwintering strategies and rest behavior on walls and how your study enters in that dinamic.*

**Response 9:** Thank you for your interesting insight. However, this paper does not evaluate temperature preference with respect to overwintering. WCSB overwinters in litters, bark crevices, buildings, etc. It is likely that WCSB selects sites with small daily temperature differences, but we have no information on this at this time.
