# Supplementary material for: Evaluation of thermoregulation of different pine organs in early spring and estimation of heat reward for the western conifer seed bug (Leptoglossus occidentalis) on male cones
Source: PLoS One. 2022 Aug 4;17(8):e0272565. doi: 10.1371/journal.pone.0272565 (PMC9352051; doi:10.1371/journal.pone.0272565)
Supplement: Attachment — Submitted filename: PLOS_Response to Reviewers_2.docx [file pone.0272565.s007.docx]

# Response to Reviewers.

Dear Reviewers,

Thank you very much for the careful review of our submitted manuscript entitled " Evaluation of thermoregulation of different pine organs in early spring and estimation of heat reward for the western conifer seed bug (*Leptoglossus occidentalis*) on male cones ". Your feedback and the detailed comments have helped us to substantially improve the manuscript.

*Academic Editor*

*Please review your reference list to ensure that it is complete and correct. If you have cited papers that have been retracted, please include the rationale for doing so in the manuscript text, or remove these references and replace them with relevant current references. Any changes to the reference list should be mentioned in the rebuttal letter that accompanies your revised manuscript. If you need to cite a retracted article, indicate the article’s retracted status in the References list and also include a citation and full reference for the retraction notice.*

**Response 1:** We reconfirmed all reference and modified the description format of some citations.

*Additional Editor Comments:*

*Line 114 - insert "a" before "heat"*

*Line 114 - insert "a" before "sunny"*

*Line 121 - replace "test" with "testing"*

*Line 123 - insert "two" between "using" and "dead WCSBs"*

*Line 131 - replace "were oriented" with "was oriented"*

*Line 158 - replace "below" with "following"*

*Line 169 - insert "the" before "Stefan-Boltzmann"*

*Line 187 - replace "below" with "the following"*

*Lines 213-215 - For readers' benefit, you might indicate the approximate wavelength range for red (~620 - 750 nm) and green (~500 - 565 nm)*

*Line 216 - replace "in" with "at"*

*Line 223 - insert "the" before "PVC" (you'll need to do this twice on line 223)*

*Line 248 - insert "the" before "PVC" and replace "pipe" with "tube" (for the sake of consistency)*

*Line 275 - the legend in Fig 10 says "PVC pipe", I recommend changing to "PVC tube" for consistency*

*Line 276 - replace "squire" with "square"*

*Line 303 - delete extra space between "male" and "cones"*

*Line 305 - delete "heat" (it's duplicated)*

*Line 310 - change "female cone" to "female cones"*

*Line 315 - rewrite: "...and female cones [38, 39], the conspicuity of WCSB on female cones [40] and so on."*

*Line 316 - replace "conclude" with "show definitively"*

*Line 320 - replace "light" with "terms"*

*Lines 320-321 - replace "and those can be same as" with "which is similarly true of"*

**Response 2:** We have adopted and corrected all of the revised comments that you have indicated.
