# Supplementary material for: Evaluation of thermoregulation of different pine organs in early spring and estimation of heat reward for the western conifer seed bug (Leptoglossus occidentalis) on male cones
Source: PLoS One. 2022 Aug 4;17(8):e0272565. doi: 10.1371/journal.pone.0272565 (PMC9352051; doi:10.1371/journal.pone.0272565)
Supplement: Peer review history

# Peer review: Decision Letter 0

**DOI:** 10.1371/journal.pone.0272565.r001

**Authors:** Frank H. Koch

8 Jun 2022

PONE-D-22-12856Evaluation of thermoregulation of different pine organs in early spring and estimation of heat reward for the western conifer seed bug (Leptoglossus occidentalis) on male flowersPLOS ONE

Dear Dr. Kume,

Thank you for submitting your manuscript to PLOS ONE. After careful consideration, we feel that it has merit but does not fully meet PLOS ONE’s publication criteria as it currently stands. Therefore, we invite you to submit a revised version of the manuscript that addresses the points raised during the review process.

Based on comments from the reviewers as well as my own reading, I believe your manuscript is not quite suitable for publication at this point. However, I think it can get there with some relatively minor changes. I have attached an annotated version of your manuscript PDF with some editorial suggestions. Although I recognize that some other researchers have called immature pine cones "flowers", I discourage you from using this terminology since pines (as gymnosperms) are not flowering plants. It could be confusing to some readers. If you choose to keep the term flowers, you will need to define it for your audience.

Overall, I find myself in agreement with Reviewer 1: the conclusion that WCSB has a thermal preference for male cones/flowers is speculative. It could be true but there are other potential explanations for this preference that are just as plausible, including male cones/flowers having greater nutritional value or a more attractive suite of volatiles than female cones/flowers. Data on nutrients in male vs. female cones/flowers would be an excellent addition, but at the least, you should present and discuss some alternative explanations.

Please submit your revised manuscript by Jul 23 2022 11:59PM. If you will need more time than this to complete your revisions, please reply to this message or contact the journal office at plosone@plos.org. When you're ready to submit your revision, log on to https://www.editorialmanager.com/pone/ and select the 'Submissions Needing Revision' folder to locate your manuscript file.

Please include the following items when submitting your revised manuscript:

- A rebuttal letter that responds to each point raised by the academic editor and reviewer(s). You should upload this letter as a separate file labeled 'Response to Reviewers'.
- A marked-up copy of your manuscript that highlights changes made to the original version. You should upload this as a separate file labeled 'Revised Manuscript with Track Changes'.
- An unmarked version of your revised paper without tracked changes. You should upload this as a separate file labeled 'Manuscript'.

If you would like to make changes to your financial disclosure, please include your updated statement in your cover letter. Guidelines for resubmitting your figure files are available below the reviewer comments at the end of this letter.

If applicable, we recommend that you deposit your laboratory protocols in protocols.io to enhance the reproducibility of your results. Protocols.io assigns your protocol its own identifier (DOI) so that it can be cited independently in the future. For instructions see: https://journals.plos.org/plosone/s/submission-guidelines#loc-laboratory-protocols. Additionally, PLOS ONE offers an option for publishing peer-reviewed Lab Protocol articles, which describe protocols hosted on protocols.io. Read more information on sharing protocols at https://plos.org/protocols?utm_medium=editorial-email&utm_source=authorletters&utm_campaign=protocols.

We look forward to receiving your revised manuscript.

Kind regards,

Frank H. Koch, PhD

Academic Editor

PLOS ONE

Journal Requirements:

When submitting your revision, we need you to address these additional requirements.

1. Please ensure that your manuscript meets PLOS ONE's style requirements, including those for file naming. The PLOS ONE style templates can be found at 

https://journals.plos.org/plosone/s/file?id=wjVg/PLOSOne_formatting_sample_main_body.pdf and 

https://journals.plos.org/plosone/s/file?id=ba62/PLOSOne_formatting_sample_title_authors_affiliations.pdf

2. Please review your reference list to ensure that it is complete and correct. If you have cited papers that have been retracted, please include the rationale for doing so in the manuscript text, or remove these references and replace them with relevant current references. Any changes to the reference list should be mentioned in the rebuttal letter that accompanies your revised manuscript. If you need to cite a retracted article, indicate the article’s retracted status in the References list and also include a citation and full reference for the retraction notice.

3. We note that the grant information you provided in the ‘Funding Information’ and ‘Financial Disclosure’ sections do not match. 

When you resubmit, please ensure that you provide the correct grant numbers for the awards you received for your study in the ‘Funding Information’ section.

4. Thank you for stating the following in the Acknowledgments Section of your manuscript: 

"This study was partly supported by JSPS KAKENHI Grant Number JP18H02511. We thank Mallory Eckstut, PhD, from Edanz (https://jp.edanz.com/ac) for editing a draft of this manuscript."

We note that you have provided funding information that is not currently declared in your Funding Statement. However, funding information should not appear in the Acknowledgments section or other areas of your manuscript. We will only publish funding information present in the Funding Statement section of the online submission form. 

Please remove any funding-related text from the manuscript and let us know how you would like to update your Funding Statement. Currently, your Funding Statement reads as follows: 

"The authors received no specific funding for this work."

Please include your amended statements within your cover letter; we will change the online submission form on your behalf.

Additional Editor Comments:

Please see the attached PDF.

[Note: HTML markup is below. Please do not edit.]

Reviewers' comments:

Reviewer's Responses to Questions

**Comments to the Author**

1. Is the manuscript technically sound, and do the data support the conclusions?

The manuscript must describe a technically sound piece of scientific research with data that supports the conclusions. Experiments must have been conducted rigorously, with appropriate controls, replication, and sample sizes. The conclusions must be drawn appropriately based on the data presented. 

Reviewer #1: Yes

Reviewer #2: Yes

**********

2. Has the statistical analysis been performed appropriately and rigorously? 

Reviewer #1: I Don't Know

Reviewer #2: Yes

**********

3. Have the authors made all data underlying the findings in their manuscript fully available?

The PLOS Data policy requires authors to make all data underlying the findings described in their manuscript fully available without restriction, with rare exception (please refer to the Data Availability Statement in the manuscript PDF file). The data should be provided as part of the manuscript or its supporting information, or deposited to a public repository. For example, in addition to summary statistics, the data points behind means, medians and variance measures should be available. If there are restrictions on publicly sharing data—e.g. participant privacy or use of data from a third party—those must be specified.

Reviewer #1: Yes

Reviewer #2: Yes

**********

4. Is the manuscript presented in an intelligible fashion and written in standard English?

PLOS ONE does not copyedit accepted manuscripts, so the language in submitted articles must be clear, correct, and unambiguous. Any typographical or grammatical errors should be corrected at revision, so please note any specific errors here.

Reviewer #1: Yes

Reviewer #2: Yes

**********

5. Review Comments to the Author

Please use the space provided to explain your answers to the questions above. You may also include additional comments for the author, including concerns about dual publication, research ethics, or publication ethics. (Please upload your review as an attachment if it exceeds 20,000 characters)

Reviewer #1: The manuscript presents original data which could explain a preference of WCSB for pine male flowers in early spring. Based on field observations indicating that WCSB prefers pine male flowers to feed, the authors estimated the temperature of male flowers, female flowers, and pine needles, to support the hypothesis of a thermal preference for this microhabitat. The experiments and analyses have a high technical standard and are well described. The article is well written.

The main drawback of the Discussion is that other alternative hypotheses for a preference for male flowers should be presented and discussed. In particular, male flowers’ nutritional value, such as sugars, and N content could be also more favorable for the bug. This could be discussed by presenting data on nutritional values or at least presenting this hypothesis. Also, volatiles released by male flowers and female flowers may be very dissimilar.

Other comments:

Line 48 – Add the information about this is the adult stage – adults WCSB

Line 110 – I did not follow this part - Do you refer to dead WCSB?

Lines 121-124 – How many bugs were tested for each treatment?

Lines 312-313 – I found this part of the conclusion speculative. The authors do not have enough information to say the bugs receive significant heat just by sucking on male flowers rather than on female flowers. The results provided by the authors mainly demonstrate that by staying in male flowers the bugs may receive more heat from this microhabitat which may elevate their body temperature above air temperature. What is ingested and the respective energy budget, including the metabolic heat produced, are completely out of the scope of this work. In fact, should be discussed whether the nutritional value and sugar content of male flowers and female flowers also differ.

Reviewer #2: In general a simple but nice paper providing data on a worldwide pest. It can be improved better stating the objectives of the work, improving introduction in terms of invasiveness of WCSB and economic damage. Also I´d like to see discuss a bit on overwintering strategies and rest behavior on walls and how your study enters in that dinamic.

**********

6. PLOS authors have the option to publish the peer review history of their article (what does this mean?). If published, this will include your full peer review and any attached files.

If you choose “no”, your identity will remain anonymous but your review may still be made public.

**Do you want your identity to be public for this peer review?** For information about this choice, including consent withdrawal, please see our Privacy Policy.

Reviewer #1: No

Reviewer #2: No

**********

[NOTE: If reviewer comments were submitted as an attachment file, they will be attached to this email and accessible via the submission site. Please log into your account, locate the manuscript record, and check for the action link "View Attachments". If this link does not appear, there are no attachment files.]

While revising your submission, please upload your figure files to the Preflight Analysis and Conversion Engine (PACE) digital diagnostic tool, https://pacev2.apexcovantage.com/. PACE helps ensure that figures meet PLOS requirements. To use PACE, you must first register as a user. Registration is free. Then, login and navigate to the UPLOAD tab, where you will find detailed instructions on how to use the tool. If you encounter any issues or have any questions when using PACE, please email PLOS at figures@plos.org. Please note that Supporting Information files do not need this step.

Attachment Submitted filename: PONE-D-22-12856_fhk.pdf Click here for additional data file.

# Author comment: Author response to Decision Letter 0

**DOI:** 10.1371/journal.pone.0272565.r002

28 Jun 2022

Dear Reviewers,

Thank you very much for the careful review of our submitted manuscript entitled " Evaluation of thermoregulation of different pine organs in early spring and estimation of heat reward for the western conifer seed bug (Leptoglossus occidentalis) on male flower ". Your feedback and the detailed comments have helped us to substantially improve the manuscript. 

Academic Editor

I have attached an annotated version of your manuscript PDF with some editorial suggestions. Although I recognize that some other researchers have called immature pine cones "flowers", I discourage you from using this terminology since pines (as gymnosperms) are not flowering plants. It could be confusing to some readers. If you choose to keep the term flowers, you will need to define it for your audience.

Response 1: Thank you for your kind suggestions. In particular, it was very helpful for us to correct the English expressions. The colors used in the figures have been completely changed. The expression of reproductive organs has been unified to "cones". As a result, the title was also changed. “Evaluation of thermoregulation of different pine organs in early spring and estimation of heat reward for the western conifer seed bug (Leptoglossus occidentalis) on male cones”

Overall, I find myself in agreement with Reviewer 1: the conclusion that WCSB has a thermal preference for male cones/flowers is speculative. It could be true but there are other potential explanations for this preference that are just as plausible, including male cones/flowers having greater nutritional value or a more attractive suite of volatiles than female cones/flowers. Data on nutrients in male vs. female cones/flowers would be an excellent addition, but at the least, you should present and discuss some alternative explanations.

Response 2: We fully agree with these opinions. In fact, there are many possibilities and we are still testing them. We have added additional information and discussions from lines 313 to 323. 

Reviewer #1: The manuscript presents original data which could explain a preference of WCSB for pine male flowers in early spring. Based on field observations indicating that WCSB prefers pine male flowers to feed, the authors estimated the temperature of male flowers, female flowers, and pine needles, to support the hypothesis of a thermal preference for this microhabitat. The experiments and analyses have a high technical standard and are well described. The article is well written.

The main drawback of the Discussion is that other alternative hypotheses for a preference for male flowers should be presented and discussed. In particular, male flowers’ nutritional value, such as sugars, and N content could be also more favorable for the bug. This could be discussed by presenting data on nutritional values or at least presenting this hypothesis. Also, volatiles released by male flowers and female flowers may be very dissimilar. 

Response 3: Thank you for evaluating our research. As mentioned in Response2, we have added a discussion about the alternative hypotheses. 

Other comments:

Line 48 – Add the information about this is the adult stage – adults WCSB

Response 4: Thank you for the comment. I added such explanation in line 46. 

Line 110 – I did not follow this part - Do you refer to dead WCSB?

Response 5: We added the explanation of the experimental condition in line 108. 

Lines 121-124 – How many bugs were tested for each treatment?

Response 6: Thank you for the question. The sample size was two and the sentence was added in line 131.

Lines 312-313 – I found this part of the conclusion speculative. The authors do not have enough information to say the bugs receive significant heat just by sucking on male flowers rather than on female flowers. The results provided by the authors mainly demonstrate that by staying in male flowers the bugs may receive more heat from this microhabitat which may elevate their body temperature above air temperature. What is ingested and the respective energy budget, including the metabolic heat produced, are completely out of the scope of this work. In fact, should be discussed whether the nutritional value and sugar content of male flowers and female flowers also differ. 

Response 7: We fully agree with these suggestions. We changed the conclusion and added description about other hypotheses in discussion from lines 313 to 323. 

Reviewer #2: In general a simple but nice paper providing data on a worldwide pest. It can be improved better stating the objectives of the work, improving introduction in terms of invasiveness of WCSB and economic damage. 

Response 8: Thank you very much for the comment. I added the damage information in introduction from lines 42 to 44. 

Also I`d like to see discuss a bit on overwintering strategies and rest behavior on walls and how your study enters in that dinamic.

Response 9: Thank you for your interesting insight. However, this paper does not evaluate temperature preference with respect to overwintering. WCSB overwinters in litters, bark crevices, buildings, etc. It is likely that WCSB selects sites with small daily temperature differences, but we have no information on this at this time.

Attachment Submitted filename: PLOS_Response to Reviewers_1.docx Click here for additional data file.

# Editor report: Decision Letter 1

**DOI:** 10.1371/journal.pone.0272565.r003

**Authors:** Frank H. Koch

14 Jul 2022

PONE-D-22-12856R1Evaluation of thermoregulation of different pine organs in early spring and estimation of heat reward for the western conifer seed bug (Leptoglossus occidentalis) on male conesPLOS ONE

Dear Dr. Kume,

Thank you for submitting your manuscript to PLOS ONE. After careful consideration, we feel that it has merit but does not fully meet PLOS ONE’s publication criteria as it currently stands. Therefore, we invite you to submit a revised version of the manuscript that addresses the points raised during the review process.

I have reviewed the revised version of your manuscript and it is nearly suitable for publication. I have a handful of minor editorial suggestions that will help with clarity and readability. If you make these minor changes (see "Additional Editor Comments" below) I should be able to move quickly to accept your manuscript.

Please submit your revised manuscript by Aug 28 2022 11:59PM. If you will need more time than this to complete your revisions, please reply to this message or contact the journal office at plosone@plos.org. When you're ready to submit your revision, log on to https://www.editorialmanager.com/pone/ and select the 'Submissions Needing Revision' folder to locate your manuscript file.

Please include the following items when submitting your revised manuscript:

- A rebuttal letter that responds to each point raised by the academic editor and reviewer(s). You should upload this letter as a separate file labeled 'Response to Reviewers'.
- A marked-up copy of your manuscript that highlights changes made to the original version. You should upload this as a separate file labeled 'Revised Manuscript with Track Changes'.
- An unmarked version of your revised paper without tracked changes. You should upload this as a separate file labeled 'Manuscript'.

If you would like to make changes to your financial disclosure, please include your updated statement in your cover letter. Guidelines for resubmitting your figure files are available below the reviewer comments at the end of this letter.

If applicable, we recommend that you deposit your laboratory protocols in protocols.io to enhance the reproducibility of your results. Protocols.io assigns your protocol its own identifier (DOI) so that it can be cited independently in the future. For instructions see: https://journals.plos.org/plosone/s/submission-guidelines#loc-laboratory-protocols. Additionally, PLOS ONE offers an option for publishing peer-reviewed Lab Protocol articles, which describe protocols hosted on protocols.io. Read more information on sharing protocols at https://plos.org/protocols?utm_medium=editorial-email&utm_source=authorletters&utm_campaign=protocols.

We look forward to receiving your revised manuscript.

Kind regards,

Frank H. Koch, PhD

Academic Editor

PLOS ONE

Journal Requirements:

Please review your reference list to ensure that it is complete and correct. If you have cited papers that have been retracted, please include the rationale for doing so in the manuscript text, or remove these references and replace them with relevant current references. Any changes to the reference list should be mentioned in the rebuttal letter that accompanies your revised manuscript. If you need to cite a retracted article, indicate the article’s retracted status in the References list and also include a citation and full reference for the retraction notice.

Additional Editor Comments:

Line 114 - insert "a" before "heat"

Line 114 - insert "a" before "sunny"

Line 121 - replace "test" with "testing"

Line 123 - insert "two" between "using" and "dead WCSBs"

Line 131 - replace "were oriented" with "was oriented"

Line 158 - replace "below" with "following"

Line 169 - insert "the" before "Stefan-Boltzmann"

Line 187 - replace "below" with "the following"

Lines 213-215 - For readers' benefit, you might indicate the approximate wavelength range for red (~620 - 750 nm) and green (~500 - 565 nm)

Line 216 - replace "in" with "at"

Line 223 - insert "the" before "PVC" (you'll need to do this twice on line 223)

Line 248 - insert "the" before "PVC" and replace "pipe" with "tube" (for the sake of consistency)

Line 275 - the legend in Fig 10 says "PVC pipe", I recommend changing to "PVC tube" for consistency

Line 276 - replace "squire" with "square"

Line 303 - delete extra space between "male" and "cones"

Line 305 - delete "heat" (it's duplicated)

Line 310 - change "female cone" to "female cones"

Line 315 - rewrite: "...and female cones [38, 39], the conspicuity of WCSB on female cones [40] and so on."

Line 316 - replace "conclude" with "show definitively"

Line 320 - replace "light" with "terms"

Lines 320-321 - replace "and those can be same as" with "which is similarly true of"

[Note: HTML markup is below. Please do not edit.]

[NOTE: If reviewer comments were submitted as an attachment file, they will be attached to this email and accessible via the submission site. Please log into your account, locate the manuscript record, and check for the action link "View Attachments". If this link does not appear, there are no attachment files.]

While revising your submission, please upload your figure files to the Preflight Analysis and Conversion Engine (PACE) digital diagnostic tool, https://pacev2.apexcovantage.com/. PACE helps ensure that figures meet PLOS requirements. To use PACE, you must first register as a user. Registration is free. Then, login and navigate to the UPLOAD tab, where you will find detailed instructions on how to use the tool. If you encounter any issues or have any questions when using PACE, please email PLOS at figures@plos.org. Please note that Supporting Information files do not need this step.

# Author comment: Author response to Decision Letter 1

**DOI:** 10.1371/journal.pone.0272565.r004

18 Jul 2022

Response to Reviewers.

Dear Reviewers,

Thank you very much for the careful review of our submitted manuscript entitled " Evaluation of thermoregulation of different pine organs in early spring and estimation of heat reward for the western conifer seed bug (Leptoglossus occidentalis) on male cones ". Your feedback and the detailed comments have helped us to substantially improve the manuscript. 

Academic Editor

Please review your reference list to ensure that it is complete and correct. If you have cited papers that have been retracted, please include the rationale for doing so in the manuscript text, or remove these references and replace them with relevant current references. Any changes to the reference list should be mentioned in the rebuttal letter that accompanies your revised manuscript. If you need to cite a retracted article, indicate the article’s retracted status in the References list and also include a citation and full reference for the retraction notice.

Response 1: We reconfirmed all reference and modified the description format of some citations. 

Additional Editor Comments:

Line 114 - insert "a" before "heat"

Line 114 - insert "a" before "sunny"

Line 121 - replace "test" with "testing"

Line 123 - insert "two" between "using" and "dead WCSBs"

Line 131 - replace "were oriented" with "was oriented"

Line 158 - replace "below" with "following"

Line 169 - insert "the" before "Stefan-Boltzmann"

Line 187 - replace "below" with "the following"

Lines 213-215 - For readers' benefit, you might indicate the approximate wavelength range for red (~620 - 750 nm) and green (~500 - 565 nm)

Line 216 - replace "in" with "at"

Line 223 - insert "the" before "PVC" (you'll need to do this twice on line 223)

Line 248 - insert "the" before "PVC" and replace "pipe" with "tube" (for the sake of consistency)

Line 275 - the legend in Fig 10 says "PVC pipe", I recommend changing to "PVC tube" for consistency

Line 276 - replace "squire" with "square"

Line 303 - delete extra space between "male" and "cones"

Line 305 - delete "heat" (it's duplicated)

Line 310 - change "female cone" to "female cones"

Line 315 - rewrite: "...and female cones [38, 39], the conspicuity of WCSB on female cones [40] and so on."

Line 316 - replace "conclude" with "show definitively"

Line 320 - replace "light" with "terms"

Lines 320-321 - replace "and those can be same as" with "which is similarly true of"

Response 2: We have adopted and corrected all of the revised comments that you have indicated.

Attachment Submitted filename: PLOS_Response to Reviewers_2.docx Click here for additional data file.

# Editor report: Decision Letter 2

**DOI:** 10.1371/journal.pone.0272565.r005

**Authors:** Frank H. Koch

22 Jul 2022

Evaluation of thermoregulation of different pine organs in early spring and estimation of heat reward for the western conifer seed bug (Leptoglossus occidentalis) on male cones

PONE-D-22-12856R2

Dear Dr. Kume,

We’re pleased to inform you that your manuscript has been judged scientifically suitable for publication and will be formally accepted for publication once it meets all outstanding technical requirements.

Within one week, you’ll receive an e-mail detailing the required amendments. When these have been addressed, you’ll receive a formal acceptance letter and your manuscript will be scheduled for publication.

An invoice for payment will follow shortly after the formal acceptance. To ensure an efficient process, please log into Editorial Manager at http://www.editorialmanager.com/pone/, click the 'Update My Information' link at the top of the page, and double check that your user information is up-to-date. If you have any billing related questions, please contact our Author Billing department directly at authorbilling@plos.org.

If your institution or institutions have a press office, please notify them about your upcoming paper to help maximize its impact. If they’ll be preparing press materials, please inform our press team as soon as possible -- no later than 48 hours after receiving the formal acceptance. Your manuscript will remain under strict press embargo until 2 pm Eastern Time on the date of publication. For more information, please contact onepress@plos.org.

Kind regards,

Frank H. Koch, PhD

Academic Editor

PLOS ONE

Additional Editor Comments (optional):

Thank you for completing one last set of revisions. Your manuscript is acceptable for publication.

Reviewers' comments:

# Editor report: Acceptance letter

**DOI:** 10.1371/journal.pone.0272565.r006

**Authors:** Frank H. Koch

26 Jul 2022

PONE-D-22-12856R2 

Evaluation of thermoregulation of different pine organs in early spring and estimation of heat reward for the western conifer seed bug *(Leptoglossus occidentalis)* on male cones

Dear Dr. Kume:

I'm pleased to inform you that your manuscript has been deemed suitable for publication in PLOS ONE. Congratulations! Your manuscript is now with our production department. 

If your institution or institutions have a press office, please let them know about your upcoming paper now to help maximize its impact. If they'll be preparing press materials, please inform our press team within the next 48 hours. Your manuscript will remain under strict press embargo until 2 pm Eastern Time on the date of publication. For more information please contact onepress@plos.org.

If we can help with anything else, please email us at plosone@plos.org. 

Thank you for submitting your work to PLOS ONE and supporting open access. 

Kind regards, 

PLOS ONE Editorial Office Staff

on behalf of

Dr. Frank H. Koch 

Academic Editor

PLOS ONE
